# Loss-of-function mutations of the TIE1 receptor tyrosine kinase cause late-onset primary lymphedema

**DOI:** 10.1172/JCI173586

**Published:** 2024-05-30

**Authors:** Pascal Brouillard, Aino Murtomäki, Veli-Matti Leppänen, Marko Hyytiäinen, Sandrine Mestre, Lucas Potier, Laurence M. Boon, Nicole Revencu, Arin Greene, Andrey Anisimov, Miia H. Salo, Reetta Hinttala, Lauri Eklund, Isabelle Quéré, Kari Alitalo, Miikka Vikkula

**Affiliations:** 1 Human Molecular Genetics, de Duve Institute, University of Louvain, Brussels, Belgium.; 2 Wihuri Research Institute, Biomedicum Helsinki, Helsinki, Finland.; 3 Translational Cancer Medicine Program, Faculty of Medicine, University of Helsinki, Helsinki, Finland.; 4 Department of Vascular Medicine, Centre de Référence des Maladies Lymphatiques et Vasculaires Rares, Inserm IDESP, CHU Montpellier, Université de Montpellier, Montpellier, France.; 5 Center for Vascular Anomalies, Division of Plastic Surgery, Cliniques Universitaires Saint-Luc, University of Louvain, VASCERN-VASCA Reference Centre, Brussels, Belgium.; 6 Center for Human Genetics, Cliniques Universitaires Saint-Luc, University of Louvain, Brussels, Belgium.; 7 Department of Plastic and Oral Surgery, Lymphedema Program, Boston Children’s Hospital, Harvard Medical School, Boston, Massachusetts, USA.; 8 Biocenter Oulu, Research Unit of Clinical Medicine and Medical Research Center Oulu, University of Oulu and Oulu University Hospital, Oulu, Finland.; 9 Oulu Center for Cell-Matrix Research, Faculty of Biochemistry and Molecular Medicine, Biocenter Oulu, University of Oulu, Oulu, Finland.; 10 WELBIO department, WEL Research Institute, Wavre, Belgium.

**Keywords:** Genetics, Vascular biology, Genetic diseases, Lymph, Mouse models

## Abstract

Primary lymphedema (PL), characterized by tissue swelling, fat accumulation, and fibrosis, results from defects in lymphatic vessels or valves caused by mutations in genes involved in development, maturation, and function of the lymphatic vascular system. Pathogenic variants in various genes have been identified in about 30% of PL cases. By screening of a cohort of 755 individuals with PL, we identified two *TIE1* (tyrosine kinase with immunoglobulin- and epidermal growth factor–like domains 1) missense variants and one truncating variant, all predicted to be pathogenic by bioinformatic algorithms. The TIE1 receptor, in complex with TIE2, binds angiopoietins to regulate the formation and remodeling of blood and lymphatic vessels. The premature stop codon mutant encoded an inactive truncated extracellular TIE1 fragment with decreased mRNA stability, and the amino acid substitutions led to decreased TIE1 signaling activity. By reproducing the two missense variants in mouse *Tie1* via CRISPR/Cas9, we showed that both cause edema and are lethal in homozygous mice. Thus, our results indicate that TIE1 loss-of-function variants can cause lymphatic dysfunction in patients. Together with our earlier demonstration that ANGPT2 loss-of-function mutations can also cause PL, our results emphasize the important role of the ANGPT2/TIE1 pathway in lymphatic function.

## Abstract 2

Genetic, structural, biochemical, and functional analysis of endothelial receptor tyrosine kinase TIE1 variants in patients reveals that TIE1 loss-of-function alleles cause late-onset primary lymphedema.

## Graphical abstract

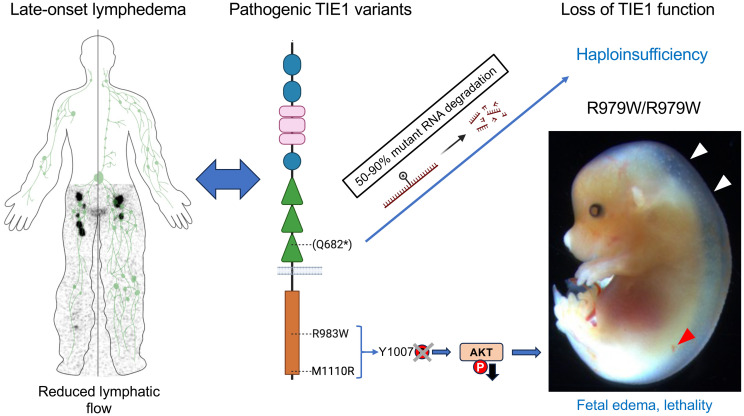

## Introduction

The pathogenesis of primary lymphedema (PL) results from defective development and/or function of the lymphatic system (ref. 1; reviewed in refs. 2–5). PL is characterized by accumulation of interstitial lymph, mostly in the limbs, but PL can also affect other parts of the body, and with time, PL results in increased fat accumulation, inflammation, and fibrosis. PL can develop in utero and be congenital, such as in Nonne-Milroy disease caused by VEGFR3 (*FLT4* gene) mutations (6, 7), but it can also appear later in life, during puberty or adulthood, such as in patients with *EPHB4*-related PL (8). In lymphoscintigraphy, the lymphatic system in PL seems most often hypoplastic, but it can also be hyperplastic.

Thirty-three genes or loci have been shown to cause PL, as evidenced by cosegregation of variants in several pedigrees, and/or by results from complementary functional in vitro and/or in vivo analysis (2, 9, 10). Variants in another 22 genes have been suggested to be causal, but without formal proof (2, 11). Originally centered around the VEGFC/VEGFR3 signaling pathway (1, 7, 12), mutations in additional ligand-receptor families have emerged as causes of PL.

The ANGPT/TIE ligand-receptor signaling pathway is necessary for blood and lymphatic vessel remodeling during embryonic and postnatal development, and for maintenance of the mature vasculature (13–15). Angiopoietin-1 (ANGPT1) is the primary multimeric agonist that binds to the TIE2 (tyrosine kinase with immunoglobulin- and epidermal growth factor–like domains 2) extracellular domain, causing receptor clustering and activation (16–18). Deletion of the mouse *Angpt1* gene is lethal in embryos (17). The second ligand, ANGPT2, mainly produced by endothelial cells, functions as an autocrine TIE2 antagonist or agonist, depending on the context (19–21). *Angpt2* gene deletion is compatible with embryonic vascular development but subsequently results in abnormal angiogenic remodeling and defective lymphatic vessels (22). Deletion of the third angiopoietin (*Angpt4*) in mice results in defective venous maturation in the retina (23), and combined deletion of *Angpt2* and *Angpt4* in a hypomorphic Schlemm’s canal (lymphatic-like vessel in the anterior part of the eye) and glaucomatous changes in the eyes (24).

The function of TIE1, discovered in 1992, is still poorly understood (25). TIE1 does not bind angiopoietins, yet both TIE1 and TIE2 are required for the development of embryonic blood and lymphatic vessels in mice (26–29). Vasculature in *Tie1*-deficient embryos fails to mature, resulting in embryonic death during late gestation (30, 31). Notably, homozygous deletion of mouse *Tie1* was shown to impair the development of lymphatic vessels and Schlemm’s canal, resulting in elevated intraocular pressure (26, 32). In adult mice, TIE1 regulates TIE2 in angiogenic tip cells and sustains TIE2 signaling in the remodeling stalk cells in the vasculature (33). TIE1 and TIE2 are also involved in vascular pathologies, including atherosclerosis and tumor angiogenesis (34–37).

TIE1 is capable of modulating TIE2 activity in a context-dependent manner by forming TIE1/TIE2 heteromers. Angiopoietin ligands and designed angiopoietin variants, such as pentameric Comp-ANGPT1, induce TIE1 phosphorylation in a TIE2-dependent manner (18, 38–40). Binding of an agonistic ligand to TIE2 activates a number of intracellular signaling pathways, notably the PI3K (phosphoinositide 3-kinase)/AKT, MAPK (mitogen-activated protein kinase), and DOK-R pathways (41–43). In contrast, the contribution of TIE1 in ANGPT signaling is incompletely understood. TIE1 is essential for the agonistic activity of ANGPT1 and the autocrine activity of ANGPT2 on TIE2, as its deletion from endothelium in adult mice reduced TIE2 phosphorylation and downstream AKT activation (40). Cleavage of the ectodomain of TIE1 promoted by inflammation was associated with a switch of ANGPT2 from a TIE2 agonist to an antagonist (40, 44). Furthermore, AKT activation mediated by autocrine ANGPT2 and TIE1 was required for expression of VEGFR3 on lymphatic endothelial cell surface, as well as for VEGFC-induced lymphangiogenesis in adult mice (45).

Genetic variants of components belonging to the ANGPT/TIE receptor signaling pathway have been associated with several diseases. Gain-of-function mutations in TIE2 (gene *TEK*) underlie inherited venous malformations (VMCM) (46), whereas TIE2 loss-of-function mutations cause primary congenital glaucoma (PCG) (47). Somatic gain-of-function TIE2 mutations are associated with sporadic venous malformations, blue rubber bleb nevus syndrome (48), and multifocal sporadic venous malformations (48, 49). Pathogenic *ANGPT1* variants have been linked to hereditary angioedema and primary glaucoma (50, 51). We recently demonstrated that a heterozygous *ANGPT2* deletion and 3 pathogenic variants that result in loss of function of ANGPT2 cause autosomal dominant PL (21), whereas another heterozygous ANGPT2 amino acid substitution caused instead a gain of function that led to cutaneous lymphatic hyperplasia (21). More recently, we also linked a biallelic *ANGPT2* loss-of-function variant to severe early-onset hydrops fetalis (52).

Here, we identify three *TIE1* variants predicted to be pathogenic and associated with late-onset PL among a cohort of 755 PL patients. We demonstrate how these variants alter the TIE1 structure and lead to TIE1 dysfunction in vitro and in vivo. Our findings underscore TIE1 loss of function as a mechanism leading to PL and highlight the important role that ANGPT2/TIE1 signaling plays in lymphatic vessel function.

## Results

### Identification of three rare TIE1 variants

**Figure 1:**
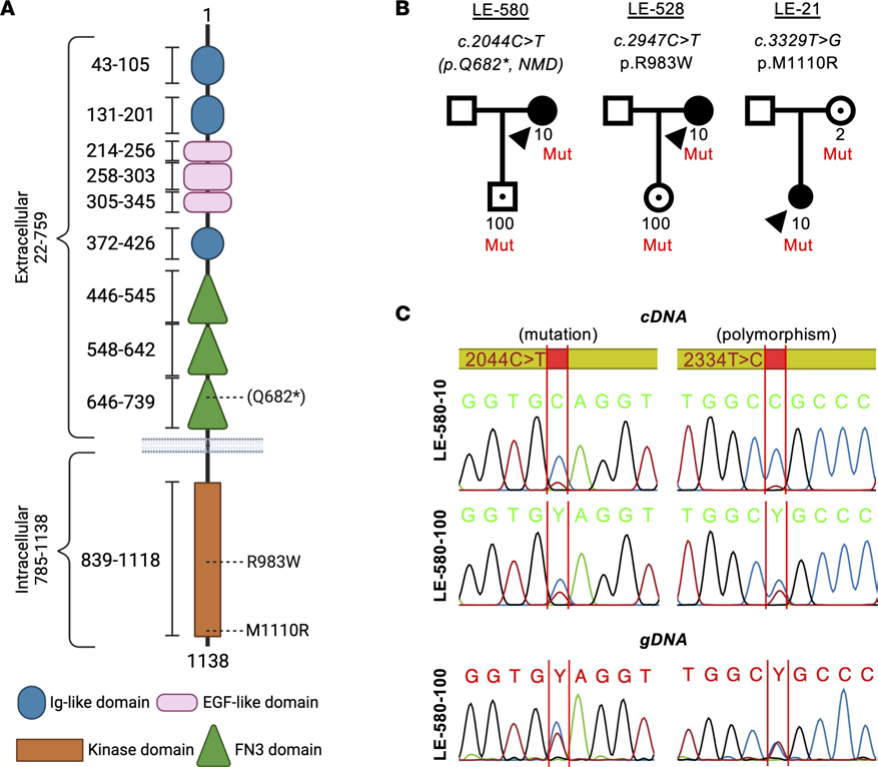
Identification of three *TIE1* variants in primary lymphedema patients. (**A**) Schematic structure of TIE1 shown with its domains and the variants, created with BioRender (biorender.com). (**B**) Pedigrees of the 3 families and cosegregation of the variants. Arrowheads, index patients; black symbols, affected individuals; black dots within symbols, unaffected variant carriers. Only numbered individuals were tested. (**C**) Reverse transcriptase PCR showing degradation of the alternative *TIE1* allele in LE-580-10 and partial degradation in LE-580-100, also confirmed by use of the *c.2334T>C*;p.Ala778= heterozygous polymorphism. LE-580-100 genomic DNA (gDNA) sequences show heterozygous levels of the variant peaks.

Sequencing the exomes (whole-exome sequencing) of 755 PL index patients led to the discovery of three *TIE1* variants predicted to be highly pathogenic (Table 1). These variants included 1 premature stop codon in the extracellular part of TIE1 and 2 amino acid substitutions located in the TIE1 kinase domain (Figure 1A). The variants, named according to the Human Genome Variation Society nomenclature, were extremely rare or absent in the Genome Aggregation Database (gnomAD) v3 (https://gnomad.broadinstitute.org), in the deCODE Allele Frequency Browser (deCAF; https://decaf.decode.com), and in the Regeneron Genetics Center Mexico City Prospective Study variant browser (RGC-MCPS; https://rgc-mcps.regeneron.com/home) (Table 1). The 2 missense variants were predicted to be damaging by 19 of 20 bioinformatic algorithms used by us.

### A TIE1 stop allele undergoes nonsense-mediated mRNA decay

**Figure 2:**
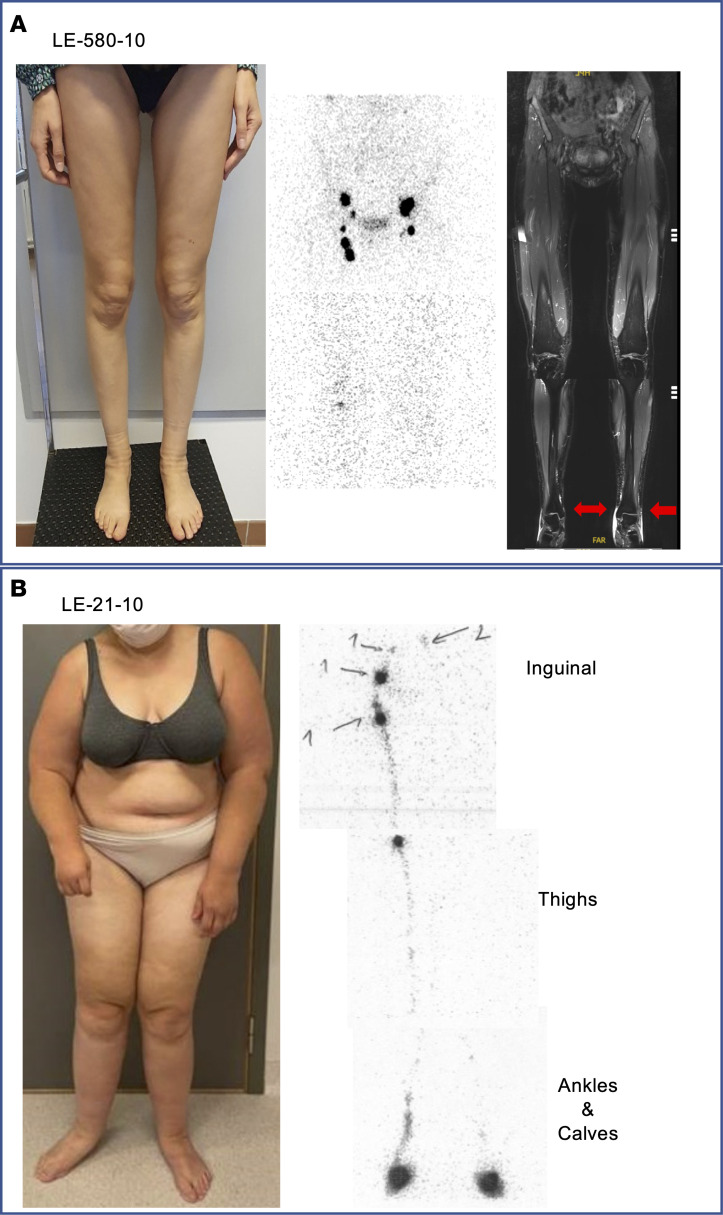
Clinical images of 2 patients with TIE1 variants. (**A**) Patient LE-580-10, with premature stop codon Q682*, has a stable clinical presentation of mild distal stage 2a lower-limb edema, according to the ISL classification. Lymphoscintigraphy (middle) shows bilateral insufficiency of lymph drainage and fewer lymph nodes, at 4 hours after injection, as well as the presence of a deep popliteal lymph node only on the right leg. The MRI image (right) shows edema (bright signal, red arrows) in the ankles, more marked on the left foot. (**B**) Photograph of patient LE-21-10 (M1110R missense variant). Lymphoscintigraphy taken 30 minutes after tracer injection reveals defective lymphatic function in the left leg.

The index patient of family LE-580 (LE-580-10; Figure 1B and Figure 2A) is a 51-year-old Portuguese woman with bilateral lower-limb lymphedema of stage 2a, according to the International Society of Lymphology (ISL) classification, without family history of PL. Her leg swelling started at 25 years of age in the whole left leg, whereas in the right leg, it involved only the foot and ankle. Her PL did not progress during pregnancy. She presented with an episode of left foot intertrigo when 41 years old. Lymphoscintigraphy showed a normal number of right inguinal nodes, but signal from the tracer increased only at 4 hours after injection, indicating delayed transport (Figure 2A, middle). Her scintigram showed also a deep popliteal node supply, indicating abnormal lymph routing. On the left leg, only a few inguinal nodes with poor activity were visible initially and at 4 hours; deep nodes or dermal backflow were not visible, underscoring strongly reduced lymphatic drainage. MRI showed edema in the ankle, which was more marked on the left side (Figure 2A, right).

The patient was found to have a heterozygous *TIE1* variant (NM_005424:*c.2044C>T*), which is extremely rare in the general population (only 4 instances in gnomAD, not in deCAF, and 8 instances in RGC-MCPS) (Table 1). This mutation encodes a premature stop codon, located in the extracellular domain of TIE1 (p.Gln682*). Any mutant TIE1 protein produced by this allele was predicted to encode only a secreted extracellular fragment of TIE1 (Figure 1A). In mRNA extracted from EBV-immortalized lymphoblasts of the patient, the variant allele was barely detectable by reverse transcriptase PCR, as compared with the WT allele, suggesting degradation of the mutant *TIE1* mRNA by nonsense-mediated decay (NMD) (Figure 1C). The 14-year-old son of the index patient (LE-580-100) is an unaffected carrier of the mutation (Figure 1B). Interestingly, in his lymphoblasts, the mutant allele (tested at the mutation site and at a heterozygous polymorphism) was only partially degraded by NMD (Figure 1C). Thus, about half of the mutant allele (~25% of total RNA) would be able to encode the truncated protein.

### Two families with TIE1 missense variants

#### Family LE-528.

The index patient of the French-Italian family LE-528 did not have family history of lymphedema. She developed PL distally in the left foot after a long flight, when she was 43 years old (Figure 1B). Her unilateral lymphedema (stage 2a) is located only in the foot, but does not involve the toes. She wears compression stockings (class 2) and self-bandages daily. She has 2 children without edema, and her lymphedema was not aggravated by her pregnancies. She had a transient ischemic attack in 2020, which led to the discovery of a large foramen ovale with an aneurysm of the interauricular septum and massive blood reflux. The defect was closed with a prosthetic device. Lymphoscintigraphy showed no drainage or nodes 4 hours after tracer injection into the left leg, whereas the right leg appeared normal (data not shown). Her lymphedema has not been analyzed by MRI.

This patient has a heterozygous NM_005424:*c.2947C>T* *TIE1* variant, reported only twice in gnomAD and 3 times in both deCAF and RGC-MCPS (Table 1). The variant amino acid (p.Arg983Trp) is located in the tyrosine kinase domain of TIE1 (R983W; Figure 1A). The same variant was transmitted to her unaffected daughter (LE-528-100), who is now 18 years old (Figure 1B). DNA from her unaffected son was not available for testing.

#### Family LE-21.

The index patient of the Belgian family LE-21 is a woman who developed PL during puberty, at the age of 16, first in the left arm, subsequently extending to all 4 limbs and to the eyelids (Figure 2B). The extent of her lymphedema has fluctuated, increasing in warm weather conditions. She is treated once a week with lymphatic draining massages and wears stockings only when the weather is warm or when her physical activity level is low. Her lymphedema is painful. As previously reported (53), lymphoscintigraphies performed at 17, 19, and 21 years old showed (a) large nodal gaps in the middle of the right inguinal lymph node cluster, as well as in lymph nodes in the left iliac external cluster and at right iliolumbar-aortic junction, and (b) decreased tracer decay at the level of the injection sites. Furthermore, (c) after phase 1, an unusual direct lymphatic vessel was found to drain into intra-abdominal lymph nodes on the left side and not to the low femoro-inguinal node on the right side (Figure 2B).

In this patient, we identified a heterozygous NM_005424:*c.3329T>G* variant in *TIE1* that leads to the replacement of methionine 1110 by an arginine (p.Met1110Arg) in the TIE1 tyrosine kinase domain (M1110R; Table 1 and Figure 1A). This variant is absent from gnomAD and RGC-MCPS and occurred 2 times in deCAF. The patient’s unaffected mother carries the same variant (Figure 1B). The index patient also suffers from Crouzon syndrome caused by a heterozygous *c.833G>T*;p.(Cys278Phe) pathogenic variant of the *FGFR2* gene (NM_022970) (53). This variant was not inherited from her mother. Her father’s DNA was not available for testing.

### Predicted structures of the two TIE1 missense proteins

We first focused on structural analysis of the 2 amino acid substitution variants, which were predicted to be pathogenic as they likely produce functionally altered TIE1 proteins. As illustrated in the protein homology model in Supplemental Figure 1 (supplemental material available online with this article; https://doi.org/10.1172/JCI173586DS1), the variant amino acid residues are located on the surface of the TIE1 kinase domain. The R983W substitution occurs at the entrance to the highly conserved ATP-binding pocket that is important for tyrosine kinase activity (Supplemental Figure 1A). Replacement of arginine with tryptophan in this location would interfere with the binding of Mg^2+^-ATP analog in a homologous position in an FGFR2 structural model, suggesting that a similar substitution in TIE1 would block ATP binding. The M1110R substitution is located in the C-terminal half of the TIE1 kinase domain, where it would produce unfavorable polar and nonpolar van der Waals interactions that result in structure-disrupting steric clashes indicated by the red discs in Supplemental Figure 1B.

### Effect of TIE1 variants on TIE1 expression and baseline phosphorylation

**Figure 3:**
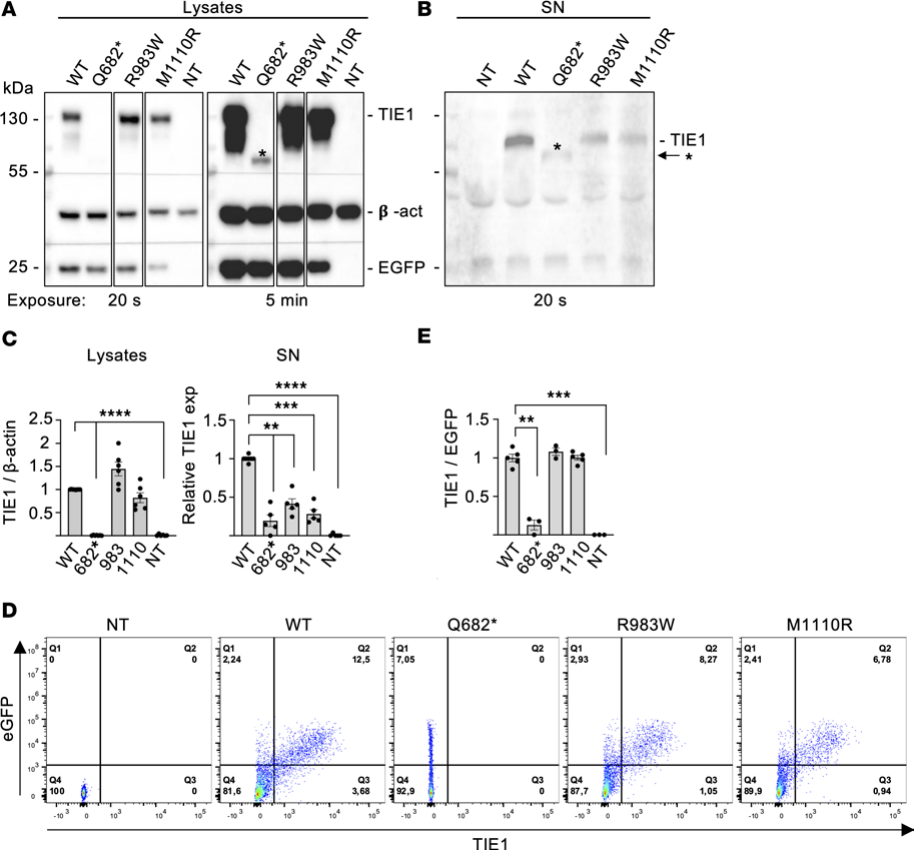
Analysis of TIE1 protein in HEK293T expressing the different TIE1 variants. (**A** and **B**) Western blotting of WT-TIE1 and the 3 variants in HEK293T cells transfected using a lentiviral vector. The truncated TIE1-Q682* variant is detected in cell lysate (**A**) only after 5 minutes of exposure, and is weak in the supernatant (**B**) after a 20-second exposure. Asterisks indicate the truncated Q682* polypeptide. The lanes were run on the same gel but were noncontiguous. (**C**) Relative TIE1 protein signals in the lysates and culture supernatants from *n* = 3 experiments like in **A** and **B**. (**D**) Flow cytometry analysis of TIE1 in transfected HEK293T cells. (**E**) Quantification of **D** from *n* = 3 experiments. Statistical significance in **C** and **E** was determined with Brown-Forsythe ANOVA with Dunnett’s post hoc test for multiple comparisons. Data shown as mean ± SEM. **P < 0.01, ***P < 0.001, ****P < 0.0001.

To analyze expression of the TIE1 variants, we transfected HEK293T cells with a plasmid that expresses the WT TIE1, or the Q682*, R983W, or M1110R variants, and then analyzed the cell lysates and culture media by Western blotting (Figure 3, A–C). WT-TIE1 was readily detected in the transfected cells after a 20-second exposure, whereas the Q682*-TIE1 protein could only be detected after a 5-minute exposure. In the supernatants from cells expressing WT-TIE1 and the 2 missense variants, we detected the shedded 100 kDa TIE1 extracellular domain produced upon ADAM17 metalloprotease cleavage between residues E749 and S750 (54), whereas in the Q682*-TIE1 supernatants, a much weaker polypeptide band of about 75 kDa was observed (Figure 3B). This was unexpected, as the GFP polypeptide and fluorescent signal produced via an IRES from the TIE1-encoding bicistronic RNA was detected in all transfected cells at the same intensity independently of the variant vector that was used, suggesting similar overall production of the TIE1 proteins. This should have led to accumulation of the truncated Q682*-TIE1 protein in the supernatant. Flow cytometry of TIE1-transfected HEK293T cells confirmed that unlike WT-, R983W-, and M1110R-TIE1, the truncated Q682*-TIE1 protein was not present on the cell surface (Figure 3, D and E). Thus, although the vector-produced RNA escaped NMD, most of the Q682*-TIE1 protein was degraded in cell culture.

**Figure 4:**
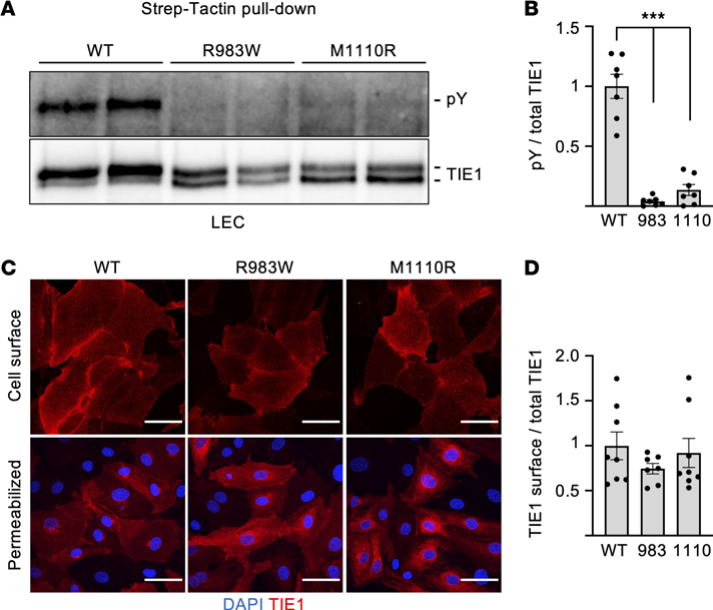
Analysis of TIE1 protein baseline phosphorylation in LECs expressing the different TIE1 variants. (**A**) Western blotting analysis of TIE1 baseline phosphorylation using the 4G10 anti-pY antibody and total TIE1 protein in LECs transduced with WT-, R983W-, or M1110R-TIE1 variant. (**B**) Quantification of phosphorylated TIE1 relative to total TIE1, from *n* = 3 independent experiments like in **A**. (**C**) Immunofluorescence staining for DAPI and TIE1 in non-permeabilized and permeabilized LECs transduced with WT-, R983W-, or M1110R-TIE1 variant. Scale bars: 100 μm. (**D**) Quantification of surface TIE1 relative to total TIE1 from 3–7 images like in **C** per group. Statistical significance in **B** and **D** was determined with Brown-Forsythe ANOVA with Dunnett’s post hoc test for multiple comparisons. Data shown as mean ± SEM. ***P < 0.001.

To analyze the function of the TIE1 missense variants, we fused both the mutant and WT-TIE1 proteins recombinantly to a C-terminal Strep-tag to distinguish them from the endogenous TIE1 in transfected endothelial cells. We then transduced retroviral expression vectors encoding the Strep-tagged proteins to lymphatic endothelial cells (LECs) and analyzed TIE1 tyrosyl-phosphorylation by Strep-Tactin purification followed by Western blotting. We found that the R983W- and M1110R-TIE1 variants were significantly less autophosphorylated than the WT-TIE1 (Figure 4, A and B).

All TIE1 proteins migrated as a polypeptide doublet in Western blots from the LEC Strep-Tactin pull-downs, in agreement with previous results (55) (Figure 4A). Short trypsin treatment of LECs followed by Western blotting of TIE1 led to loss of the 135 kDa polypeptide, but not the 125 kDa polypeptide, indicating that the former is expressed on the LEC surface whereas the latter is intracellular (Supplemental Figure 2A). Interestingly, the 125 kDa TIE1 polypeptide was more abundant in LECs expressing the R983W- and M1110R-TIE1 variants than in WT-TIE1 (Figure 4A). However, like in the FACS analysis of HEK293T cells, both variants and WT-TIE1 were expressed at similar levels on the LEC surface, as shown by TIE1 staining in intact versus permeabilized LECs (Figure 4, C and D). Further analysis of TIE1 in LEC immunoprecipitates digested by PNGase-F indicated a substantial contribution of glycans to the apparent molecular weight, and digestion with neuraminidase suggested that the 135 kDa form is produced from the 125 kDa polypeptide by addition of sialic acid (Supplemental Figure 2B).

### TIE1 variants show altered receptor phosphorylation and downstream signaling

**Figure 5:**
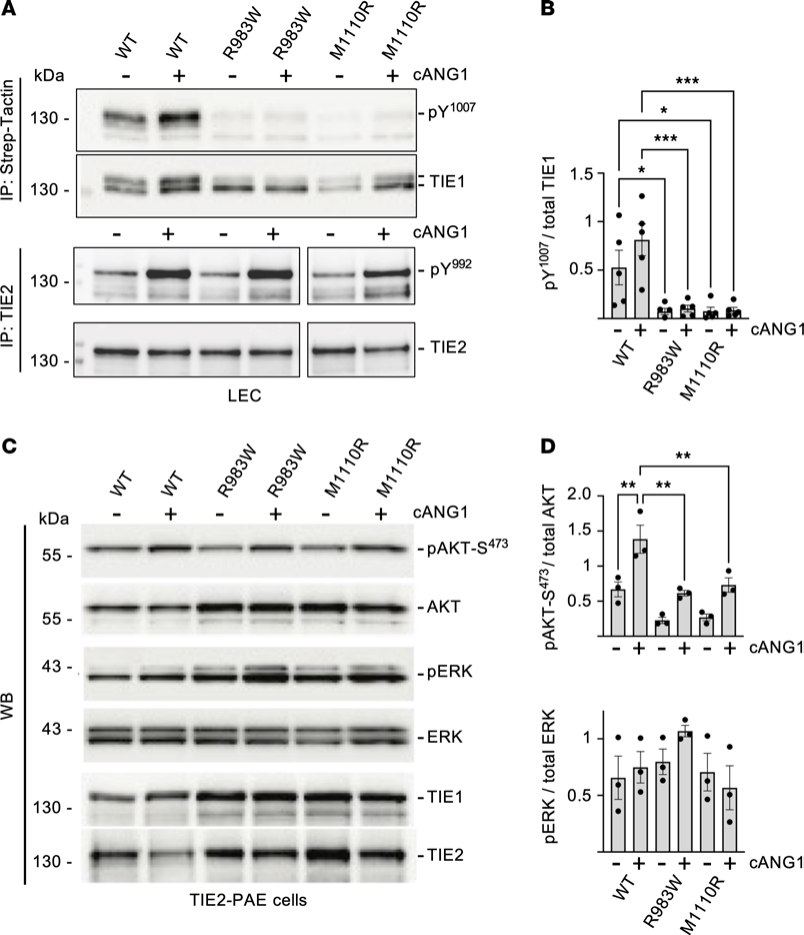
Effect of TIE1 variants R983W and M1110R on TIE1 activation and downstream signaling in endothelial cells. (**A**) Western blot analysis of TIE1 and TIE2 phosphorylation at Y1007 or Y992 residue (both pY^1007^ and pY^992^ detected by anti-pY^992^ antibody AF2720, R&D Systems) in Comp-ANGPT1–stimulated (cANG1-stimulated) LECs transduced with the WT-, R983W-, or M1110R-TIE1 variant. (**B**) Quantification of the pY^1007^/total TIE1 ratios in the samples from experiments like in **A**. Mean ± SEM, *n* = 3–5. (**C**) Western blot analysis of cANG1-stimulated TIE2-PAE cells transduced with the WT-, R983W-, or M1110R-TIE1 variant. (**D**) Quantification of phospho-AKT-S^475^/total AKT and phospho-ERK/total ERK ratios from experiments like in **C**. Data shown as mean ± SEM, *n* = 4 experiments. Statistical significance in **B** and **D** was analyzed by 1-way ANOVA with Tukey’s post hoc test for multiple comparisons. *P < 0.05, **P < 0.01, ***P < 0.001.

To analyze the effects of the TIE1 missense variants on downstream signaling, we compared Comp-ANGPT1–induced phosphorylation of WT-TIE1, R983W, and M1110R in LECs. Like endogenous TIE1 (21), the WT-TIE1 transduced in LECs was activated by Comp-ANGPT1 stimulation, as evidenced by phosphorylation of the Y1007 tyrosyl residue detected by the phosphosite-specific anti-pY992 antibody that binds to the analogous tyrosine residues also in the TIE1 receptor (Figure 5, A and B). In contrast, neither the transfected R983W-TIE1 nor M1110R-TIE1 was tyrosyl-phosphorylated in Comp-ANGPT1–stimulated LECs (Figure 5, A and B). This suggested that the variants represent mutations that decrease TIE1 activation level. To determine whether the two TIE1 loss-of-function variants also had an effect on the activity of the endogenous TIE2 receptor tyrosine kinase, we analyzed Comp-ANGPT1–induced autophosphorylation and expression of TIE2 in LECs transduced with WT-TIE1, R983W, or M1110R. We found that the two TIE1 mutants did not affect TIE2 phosphorylation (Figure 5A).

To understand in more detail the effects of the TIE1 missense mutants on downstream signaling, we established a cell culture system comparing Comp-ANGPT1–stimulated downstream signals by WT and kinase-negative TIE1 (KN; K870R corresponding to K866R in mice [ref. 56]) in TIE2-transfected porcine aortic endothelial (PAE) cells, which express low amounts of endogenous TIE2 (57, 58) (Supplemental Figure 3, A and B). Unlike WT-TIE1, the KN-TIE1 receptor led to a complete loss of baseline and Comp-ANGPT1–stimulated TIE1 autophosphorylation, as well as significantly decreased phosphorylation of the downstream AKT and ERK kinases in these cells (Supplemental Figure 3, A and B). Some Comp-ANGPT1–mediated AKT and ERK phosphorylation was observed in the KN-TIE1 cells, which could be the result of TIE2 signaling as TIE2 phosphorylation was not affected in the KN-TIE1 cells. Baseline and Comp-ANGPT1–stimulated AKT-S473 phosphorylation in TIE2-PAE cells transduced with R983W- or M1110R-TIE1 was also significantly less pronounced than that in control PAE cells transfected with WT-TIE1 (Figure 5, C and D). Interestingly, however, ERK activation was not affected by either of these mutants (Figure 5, C and D). These results indicated that both mutant TIE1 proteins had defective signaling properties in endothelial cells.

### Homozygous TIE1 missense mutants corresponding to the human variants are lethal in mice

**Figure 6:**
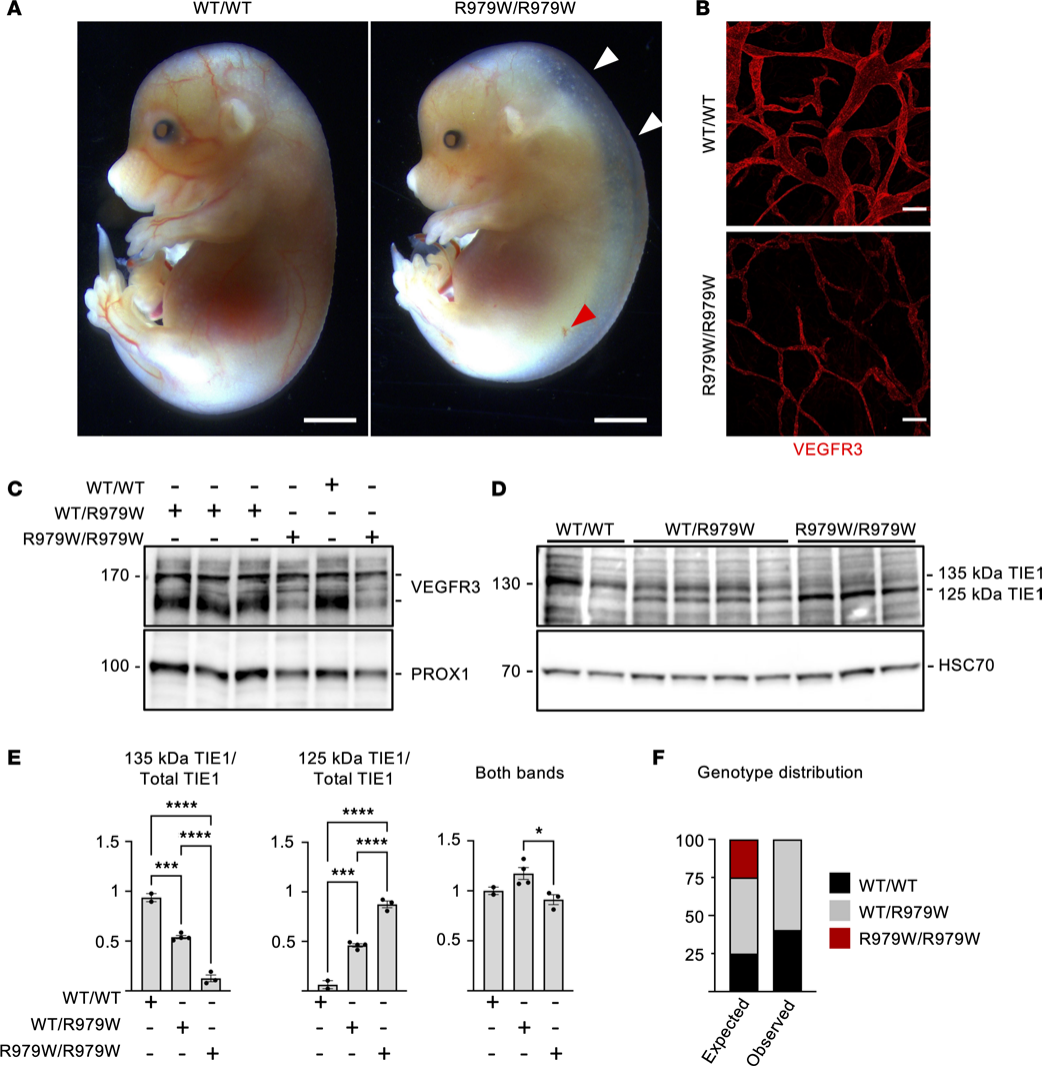
Embryos homozygous for the *Tie1^R979W^* allele display lymphatic defects and altered cell-surface/intracellular TIE1 ratio. (**A**) Macroscopic images of *Tie1^WT^* and homozygous *Tie1^R979W/R979W^* embryos at E15.5. Scale bars: 2 mm. (**B**) VEGFR3 staining of dorsal skin from WT and *Tie1^R979W/R979W^* embryos at E18.5. Scale bars: 100 μm. (**C**) Analysis of VEGFR3 polypeptides from the indicated skin lysates at E18.5. (**D**) Western blot analysis of TIE1 polypeptides from indicated lung lysates at E18.5. (**E**) Quantification of the proportions of cell-surface 135 kDa and intracellular 125 kDa TIE1 polypeptides relative to total TIE1 from the Western blots in **D**. The graph “Both bands” is the total TIE1 normalized to HSC70. *n* = 3 experiments. Statistical analysis by 1-way ANOVA with Tukey’s post hoc test for multiple comparisons. Data shown as mean ± SEM. *P < 0.05, ***P < 0.001, ****P < 0.0001. (**F**) Percentages of the indicated genotypes among pups born from heterozygous TIE1-WT/R979W matings. *n* = 8 litters (observed WT/WT *n* = 17, WT/R979W *n* = 25, R979W/R979W *n* = 0).

Mice expressing *Tie1^R979W^* and *Tie1^M1106R^* (corresponding to R983W and M1110R in humans) were generated using CRISPR/Cas9–mediated mutagenesis (Supplemental Figure 4). Only heterozygous founders were obtained, which were crossed to C57BL/6J WT mice to establish the mutant strain. Then, heterozygous mice were first crossed with each other to determine whether homozygous *Tie1^R979W/R979W^* mice were viable. At embryonic day 15.5 (E15.5), the homozygous embryos showed nuchal translucency, which is a well-established hallmark of lymphatic dysfunction (Figure 6A). Furthermore, all homozygous mutant mice died soon after birth (a total of 8 litters were analyzed; Figure 6F), as previously reported for embryos having a constitutive deletion of the *Tie1* gene (30, 31). We further analyzed the lymphatic phenotype in the homozygous embryos at E18.5 and discovered that 100% of the homozygous *Tie1^R979W/R979W^* embryos were still alive, but they appeared swollen when compared with the WT or heterozygous *Tie1^WT/R979W^* littermates and exhibited hemorrhages at the tip of the tail (Supplemental Figure 5A), resembling embryos carrying deleted or hypomorphic *Tie1* alleles (26, 31, 59). Immunofluorescence staining of tissues from the *Tie1^R979W/R979W^* embryos showed hypoplastic lymphatic vessels with immature collecting vessels and lack of valves in dorsal skin, mesentery, and intestine in comparison with their WT and heterozygous littermates (Figure 6B and Supplemental Figure 5, B–D).

As predicted by the recent finding that TIE1 function is necessary for VEGFR3 expression on the surface of lymphatic endothelial cells (45), the intensity of VEGFR3 staining in the homozygous *Tie1^R979W/R979W^* embryos was lower than that in their WT littermates (Figure 6B and Supplemental Figure 5, B–D), indicating that they have a lymphatic loss-of-function phenotype. Western blot analysis of skin lysates showed that the amount of the proteolytically cleaved, mature VEGFR3 (130 kDa) was reduced in the samples from the *Tie1^R979W/R979W^* embryos (Figure 6C), mimicking findings from *Tie1*-deleted mice (45). To determine whether the mutant TIE1 is expressed at normal rates in homozygous *Tie1^R979W/R979W^* embryos, we analyzed TIE1 proteins in embryonic lungs at E18.5 (Figure 6D). We found that the total amount of TIE1 (both bands) was quite homogeneous, but the proportion of the fully glycosylated 135 kDa cell-surface form versus the incompletely glycosylated 125 kDa intracellular form of TIE1 was consistently altered in the mutant embryos (Figure 6, D and E). The fully glycosylated TIE1 polypeptides were most prominent in Western blots from WT embryos, whereas mostly the immature intracellular form of TIE1 was detected in the homozygous *Tie1^R979W/R979W^* embryos. The heterozygous *Tie1^WT/R979W^* embryos had almost equal levels of both TIE1 forms (Figure 6, D and E). This indicated that the mutations alter posttranslational modifications and perhaps vesicular trafficking of TIE1.

We also generated mice expressing the second missense variant. Two heterozygous M1106R founders were crossed with WT C57BL/6J mice to establish mutant strains, followed by mating of the heterozygous mice with each other. About 50% of the homozygous *Tie1^M1106R/M1106R^* mice died soon after birth (Supplemental Figure 6A). Analysis of TIE1 expression in lung lysates of E18.5 *Tie1^WT^*, *Tie1^WT/M1106R^*, and *Tie1^M1106R/M1106R^* embryos by Western blotting showed again that the 125 kDa intracellular form was more abundant in *Tie1^WT/M1106R^* and *Tie1^M1106R/M1106R^* embryos than in *Tie1^WT^* embryos (Supplemental Figure 6, B and C). However, this difference was not as prominent as in the *Tie1^R979W^* embryos. Treatment of the TIE1 immunoprecipitates from adult *Tie1^WT^*, *Tie1^WT/M1106R^*, and *Tie1^M1106R/M1106R^* lung lysates with PNGase-F, which removes almost all N-linked oligosaccharides from glycoproteins, converted TIE1 polypeptides from all 3 samples into a single, approximately 100 kDa TIE1 band. Treatment with neuraminidase, which cleaves glycosidic linkages of terminal sialic acid residues in glycoproteins, reduced the molecular weight of TIE1 from about 135 kDa to 125 kDa, confirming that sialylation accounts for most of the difference between the two polypeptides (Supplemental Figure 6D). In summary, the in vivo experiments indicated that the two TIE1 missense mutants tested in mice represent loss-of-function alleles.

## Discussion

Here, we identify three rare heterozygous germline *TIE1* variants using whole-exome sequencing and detailed bioinformatic filtering in a cohort of 755 PL patients. The patients did not have a mutation in any other known PL gene. The orphan TIE1 receptor, the founding member and effector of the angiopoietin/TIE receptor tyrosine kinase pathway (25), is a crucial regulator of lymphatic development, remodeling, and lymphatic valve morphogenesis (26–28, 59). The variants that we studied affect TIE1 function variably; the 2 amino acid substitutions resulted in loss of TIE1 tyrosine kinase activity, whereas the premature stop codon resulted in haploinsufficiency. Our analyses pinpoint *TIE1* pathogenic variants as a cause of PL and underscore the major role for TIE1 in the ANGPT/TIE signaling pathway in lymphatic development and function.

All index patients developed PL in puberty or adulthood (16, 25, and 43 years old, with 28 as the mean age). This is in sharp contrast to most of the known PL-causing genetic mutations that cause hydrops fetalis and/or congenital lymphedema (2). Previously, late-onset lymphedema was associated with only a few *EPHB4* variants (8, 60). The late onset of the phenotype could explain why the 2 young mutation carriers (LE-580-100 and LE-528-100; Figure 1B) have not developed symptoms yet at 18 and 14 years old, respectively. This also suggests that in humans, TIE1 is important mainly for the maintenance of the lymphatic system, and that other factors may be needed for PL induction in the mutation carriers. In various inherited vascular anomalies, a genetic second hit is needed for lesion development (61–63). The presence of unaffected mutation carriers in dominantly inherited PL is not an exception. Mutations in *VEGFC*, for example, are only about 50% penetrant in some families (64), and in *CELSR1*, penetrance is 87% in females but only 20% in males (65).

We found only 1 affected individual per family, but also 1 unaffected mutation carrier in each. Interestingly, the transcript levels of the premature stop codon–carrying mutant allele varied between the affected (LE-580-10) and unaffected (LE-580-100) individuals of the same family. The mutant allele underwent more NMD in the affected parent than in the unaffected child, which agrees with the finding that variable NMD can explain reduced penetrance in genetic disorders (66).

### Structural and functional effects of TIE1 mutations.

TIE1 has earlier been considered as a TIE2 modulator with weak kinase activity. Yet its ligand-stimulated and TIE2-dependent downstream signaling is incompletely known. To understand the role of TIE1 in ANGPT/TIE downstream signaling, we compared the Comp-ANGPT1–stimulated signaling of WT-TIE1 to kinase-negative TIE1-K870R (56) in TIE2-PAE cells. The PAE cells show weak expression of endogenous TIE2; thus we could express TIE1 and/or TIE2 in these cells in a controlled manner (57). The TIE1-K870 residue corresponds to the TIE2-K855 that forms a salt bridge to TIE2-E872 to correctly position the α- and β-phosphates of ATP for catalysis (67). Coexpression of the kinase-negative K870R mutant of TIE1 in TIE2-PAE cells resulted in complete loss of TIE1-Y1007 phosphorylation (analogous to TIE2-Y992). This suggests that the Y1007 autophosphorylation is not TIE2 dependent, but instead TIE1 dependent: ATP needs to bind to TIE1 and the TIE1 tyrosine kinase activity leads to Y1007 phosphorylation. Furthermore, expression of the K870R mutant decreased activation of the AKT and ERK kinases despite simultaneous activation of TIE2. This indicated that TIE1 has an important function in downstream signaling of the ligand-stimulated TIE1-TIE2 complex.

The TIE1 substitutions in the kinase domain resulted in loss of TIE1 phosphorylation via different mechanisms. Structural analysis of the protein homology models indicated that the R983 residue corresponds to the highly conserved R968 residue in TIE2, which is located in the catalytic loop and makes a stabilizing salt bridge to the D979 residue in the same loop (67). An analogous R983W mutation in the highly homologous FGFR2 kinase domain complexed with an ATP analog (Protein Data Bank code 2PVF) suggests loss of an arginine-mediated stabilizing salt bridge, resulting in steric blocking of the ATP-binding site (67). Consistently with the data on the TIE1-K870R mutant, loss of ATP binding and transfer of its phosphate group in the R983W mutant resulted in loss of Y1007 autophosphorylation and reduced downstream AKT activation. However, activation of ERK by this mutant was preserved. Our structural analysis indicated also that the TIE1-M1110R mutation would lead to severe changes in protein folding close to the previously located homologous PI3K binding site in TIE1 (Y1117), which corresponds to TIE2 (Y1111) (41, 56). This likely explains the decreased AKT activation by this mutant. Loss of TIE1 phosphorylation of the M1110R mutant may reflect lack of TIE2-mediated TIE1 activation, which is required for full enhancement of TIE1 activation by Comp-ANGPT1 (38). We also found an altered ratio between the mature cell-surface and intracellular forms of TIE1 in LECs transduced with the R983W- and M1110R-TIE1 mutants and in lung lysates from mice carrying the corresponding mutations. Our results from LEC and mouse lung lysates treated with neuraminidase suggest that this results from a difference in the glycosylation of the WT versus variant TIE1 proteins. Thus, differences in intracellular processing may contribute to the reduced activity of these mutant TIE1 proteins. The reduced TIE1 variant glycosylation and trafficking to cell surface could affect angiopoietin signaling, as TIE1 has been shown to regulate TIE2 cell surface presentation in the tip cells of endothelial sprouts (33). The availability of these TIE1 mutants should facilitate further studies on TIE1 and TIE2 interactions and signaling function.

The two TIE1 amino acid substitutions led to severe phenotypes in mice homozygous for the mutations. The *Tie1^R979W/R979W^* homozygotes died perinatally, and only 50% of the *Tie1^M1106R/M1106R^* pups survived after 3 weeks of age. At E18.5, the embryos had generalized edema, underscoring a lymphatic problem. The homozygous mice carrying the R979W mutation survived until birth, whereas the majority of constitutive *Tie1*-null mice die at about E15.5 (31, 68). Homozygous pups presumably died soon after birth, as no surviving homozygous mice could be detected by genotyping of the pups at the weaning age of 3 weeks, indicating perinatal lethality. The differences in the severity of the *Tie1^R979W/R979W^* and *Tie1^M1106R/M1106R^* phenotypes indicate variability in the underlying molecular mechanisms of these two TIE1 loss-of-function mutants. The finding that a homozygous point mutation that causes inactivation of the TIE1 tyrosine kinase activity leads to lethality of mice is interesting. To our knowledge, it indicates for the first time that an active signal transducing the TIE1 tyrosine kinase is required for mouse survival. It should also be noted that although human lymphedemas often result from heterozygous pathogenic variants, most of the mutated genes cause a phenotype in mice only when fully inactivated, as was also the case in our study.

On the basis of computational analyses, three *TIE1* variants were previously suggested to be associated with PL in an Italian cohort (69). Identification of Glu1061Lys in a single patient (labeled by the authors as Glu1016Lys) was based on a TIE1 isoform transcript that is shorter than the canonical form encoding the UniProt P35590 protein. The 2 other variants, Arg481Cys (reported as Arg436Cys) and Arg1109His (as Arg1064His), segregated in 3 and 2 individuals, respectively (69). Although they have not yet been functionally validated, the late onset of the symptoms in these patients is consistent with our validated TIE1 mutations that cause loss of function. Taken together, our 3 index cases out of 755 and the 3 suggested variants reported from 235 Italian cases lead to an estimate that 6 of 990 PLs (0.6%) are explained by TIE1 variants. This prevalence is likely higher among late-onset PL.

In conclusion, we present compelling evidence that *TIE1* mutations are involved in the etiopathogenesis of primary lymphedema. The *TIE1* gene should therefore be screened among other PL genes by diagnostic services, especially in patients with a late-onset phenotype. As in the case of *ANGPT2*, our study provides a formal confirmation of the pathogenicity of the variants and elucidation of the molecular mechanisms. Furthermore, our data here point to an important role for TIE1 tyrosine kinase activity in the ANGPT/TIE signaling system, further confirming that ANGPT2 function is TIE1 dependent in the lymphatic vasculature (45). The mechanistic understanding of how the lymphatic vessel phenotypes arise is important for the planning of therapies that are tailored to target patient-specific genes and mutations.

## Methods

### Sex as a biological variant.

Sex was not considered as a biological variable. Both female and male patients and family members were included in our studies. For mouse studies as well, both female and male mice were used.

### Sequencing and variant calling.

Blood-derived DNA of index patients from 755 families with PL were subjected to whole-exome sequencing, performed at Macrogen-Europe. The samples were collected over a period of 26 years, from several centers in several countries. More than 95% were from European centers, including 60% from France and 23% from Belgium. Variants were called using our in-house-developed Highlander software (https://sites.uclouvain.be/highlander/), as described in ref. 21. Consensus prediction score (Table 1) is the sum of software tools assessing a change as damaging or with a high score, out of the 20 following predictors: Mutation Taster, FATHMM, FATHMM-XF, Polyphen2 (HDIV), Provean, SIFT4G, Mutation Assessor, MCAP, LRT, Lists2, Deogen, ClinPred, BayesDel (with MaxMAF), PrimateAI, MetaSVM, CADD, VEST, REVEL, MVP, and MutPred. A value of 400 is added for frameshifts and premature stop codons. The control population databases contained 4,454 genomes and 76,156 exomes for gnomAD v3, 150,119 individuals in deCAF, and 150,996 in RGC-MCPS, meaning altogether 381,725 samples, representing 763,450 alleles.

### RNA stability analysis.

Lymphocytes and lymphoblast-derived RNA were extracted with TriPure (Merck) or with the NucleoSpin TriPrep kit (Macherey-Nagel) and cDNA synthesis performed using the RevertAid H Minus First Strand cDNA Synthesis kit (Thermo Fisher Scientific). Primers were chosen overlapping exons 11–12 (5′-CAGACTGTCCTGAGCCTTTG-3′) and in exon 14 (5′-CAGCTTCTGCGGATGCACAC-3′) for the premature stop in family LE-580 (mutation in exon 13 and polymorphism in exon 14).

### Analysis of the TIE1 stop codon mutant in HEK293T cells.

Mutagenesis primers used on *TIE1* mRNA cloned in the gateway-derived lentiviral plasmid pFS425 (a gift from Thomas Michiels, UCLouvain) were designed using the QuikChange Primer Design tool (Agilent, https://www.agilent.com/store/primerDesignProgram.jsp) according to the recommended protocol. Plasmids were sequence-verified for the whole coding DNA sequence using Sanger sequencing. Transfections of HEK293T cells, in 6-well plates at 80% confluence, were performed using JetPEI (PolyPlus). Supernatants (1 mL) were collected 24 hours after transfection; cells were lysed using 0.5 M Tris-HCl, 1.5 M NaCl, NP-40, 0.5 M EDTA, cOmplete protease inhibitor cocktail tablet (Roche), PhosSTOP (Roche), and H_2_O. Lysates were sonicated before total protein concentration assessment using the BCA Protein Assay (Thermo Fisher Scientific 23225).

For Western blot, 12 μg of proteins (or 20 μL of supernatant) were electrophoresed on SDS-PAGE 4%–15% acrylamide gels (Bio-Rad Laboratories) for 30 minutes and transferred to PVDF membranes (Thermo Fisher Scientific). These were probed with anti-TIE1 antibody (1:3,000) (AF619, R&D Systems, raised against Ala22-Gln760), anti–β-actin (1:10,000) (A5441, Merck), and anti-GFP (1:2,000) (MA5-15256, Invitrogen) for 18 hours in TBS-Tween 0.05% buffer with 2% milk, rinsed with TBS-Tween 0.05% buffer with 2% milk, and incubated with anti-goat (1:2,000) (P0449, Agilent) for TIE1 or anti-mouse (1:10,000) (A5278, Merck) secondary antibody for β-actin and GFP. The signals were developed after 1 hour using the ECL SuperSignal West Pico PLUS Chemiluminescent Substrate (Thermo Fisher Scientific).

### Flow cytometry on HEK293T cells.

Twenty-four hours after transfection with *TIE1* lentiviral plasmid, HEK293T cells were washed with PBS supplemented with 1% FBS (F7524, Merck) and 1 mM EDTA. Then, 7.5 μg/mL of the anti-TIE1–PE (FAB619P, R&D Systems) antibody was added to cells and incubated for 30 minutes in the dark on ice. Cells were washed 3 times and resuspended in 200 mL using the same supplemented PBS buffer, and stained with DAPI solution (D3571, Thermo Fisher Scientific). Flow cytometry was performed on a BD FACSVerse at the flow cytometry and cell-sorting platform of de Duve Institute, UCLouvain, Brussels, Belgium. Data were analyzed with FlowJo software (BD Biosciences).

### Generation of viral vectors used in LECs.

The *TIE1* mutants R983W and M1110R, as well as WT-TIE1, were cloned into FUW lentivirus (Addgene 14882) and pMXs retroviral (70) vector plasmids and equipped with a C-terminal twin Strep-tag. HEK293FT and HEK293GPG packaging cells for lenti- and retroviruses, respectively, were maintained in DMEM (Lonza) and 10% FBS. The cells were transformed with Fugene 6 (Roche) in DMEM supplemented with 4.5 g/L glucose and 10% FBS. Virus-containing media were collected and changed after 72 and 96 hours, combined, and filtered. Lentiviruses were concentrated by a 2-hour ultracentrifugation at 25,000*g*, +4°C, and used to transduce LECs (Human Dermal Lymphatic Endothelial Cells, PromoCell C-12216) grown on gelatin-coated plates in Endothelial Cell Basal Medium MV 2 (ECBM, PromoCell C-22121) supplemented with SupplementPack GM MV 2 (PromoCell C-39221) and 50 ng/mL of mature VEGFC (71).

### Analysis of TIE1 and TIE2 pathway activation.

For paracrine TIE1 and TIE2 autophosphorylation, cells were starved overnight in 0.1% BSA-containing medium, followed by 1 hour of stimulation with Comp-ANGPT1, as indicated. The cells were lysed in 1% Triton 100 containing PLCLB lysis buffer (38, 72) followed by TIE1 pull-down with Strep-Tactin Sepharose (IBA Lifesciences) or by TIE2 immunoprecipitation using anti-TIE2 antibodies (AF313, R&D Systems) plus protein G–Sepharose (GE Healthsciences Ab) at +4°C. The immunocomplexes were separated in SDS-PAGE (Bio-Rad Laboratories) and transferred to PVDF membrane (Millipore) using the Trans-Blot Turbo Transfer System (Bio-Rad Laboratories). The membranes were used for immunoblotting with anti–phospho-TIE2 (pY992, AF2720, R&D Systems), anti-phosphotyrosine (05-321, clone 4G10, Cell Signaling Technology), anti-TIE1 (AF619, R&D Systems), or anti-TIE2 (AF313, R&D Systems) as primary antibodies and HRP-conjugated secondary antibodies (Dako), biotinylated secondary antibodies (Dako), or tertiary HRP-conjugated antibodies, followed by ECL detection (SuperSignal, Thermo Fisher Scientific). Phospho-AKT (T308), phospho-AKT (S473), total AKT, phospho-ERK, and total ERK were probed from Western blots with primary antibodies 9275S, 9271L, 9272, 9106S (E10), and 9102L (Cell Signaling Technology), respectively, and then imaged in Odyssey FC (LI-COR) or Azure 500 (Azure Biosystems). For reprobing, the membranes were stripped using ReBlot Plus Strong Antibody stripping buffer (Millipore). The Western blots were quantified using ImageJ (NIH) or AzureSpot Pro.

### Trypsin treatment.

LECs were grown in 6-well plates until 90% confluence. Immediately before the experiment, growth medium was removed and cell monolayers rinsed with cold PBS. Trypsin solution (Euroclone, catalog ECM0920D) was added to parallel wells (PBS was added to the control wells) and incubated for 10 minutes at +37°C. Subsequently, the cells in control wells were lysed with PLCLB lysis buffer. Detached cells from trypsin cultures were collected, growth medium with 10% FCS was added to inactivate trypsin, the cells were briefly spun down (300*g*, 5 minutes), and the cell pellet was lysed with PLCLB. Proteins in the lysates were separated in 8% SDS-PAGE, transferred to the PVDF membrane, and probed with anti-TIE1 (AF619, R&D Systems) or anti-HSC70 (sc-7298, Santa Cruz Biotechnology). All experiments were done twice with similar results. 

### Glycosidase treatment.

Mouse lung lysates were immunoprecipitated with anti-TIE1 antibodies (AF619, R&D Systems). Total N-linked glycan removal was carried out with PNGase-F (New England Biolabs P0704) using manufacturer’s reagents in the following manner: Denaturing buffer was added to washed immunoprecipitates bound to Sepharose, and samples were heated at 96°C for 10 minutes. Glycobuffer 2 and NP-40 were added, together with PNGase-F enzyme, followed by 1 hour of incubation at +37°C. For neuraminidase treatment, washed mouse lung TIE1 immunoprecipitates were mixed with reaction buffer and α2-3,6,8 Neuraminidase (New England Biolabs P0720), followed by 1 hour of incubation at +37°C. SDS-PAGE sample buffer was added to enzyme-treated reactions and analyzed by SDS-PAGE.

For LEC cell surface TIE1 glycosylation analysis, confluent LEC cultures were washed with ice-cold PBS and treated with anti-TIE1 antibodies (AF619, R&D Systems) in 3% BSA in PBS on ice for 1 hour. Cells were washed twice with ice-cold PBS and lysed in cell lysis buffer as explained in the *Analysis of TIE1 and TIE2 pathway activation* section above, and cell surface TIE1 was immunoprecipitated. Immunoprecipitates were treated with glycosidases as above and analyzed by Western blotting.

### Mouse models.

CRISPR/Cas9–based genome editing (73) was used to create knockin mouse lines carrying the mutations *Tie1*^R979W^ and *Tie1^M1106R^* corresponding to the human R983W and M1110R variants, respectively (Supplemental Figure 4). The CRISPR RNAs (crRNAs) used were designed with the CRISPR finder tool at the Wellcome Trust Sanger Institute Genome Editing website (74). In each case, a single-stranded oligodeoxynucleotide (ssODN) was used as a homology-directed repair template that carried the patient mutation, along with silent mutations to eliminate existing PAM sites, and to create a novel restriction enzyme cut site in the mouse genome to be used in genotyping.

Preassembled *Alt-R* crRNA/*Alt-R* tracrRNA/Alt-R S.p. HiFi Cas9 (Integrated DNA Technologies) ribonucleoprotein complex, together with the ssODN (Integrated DNA Technologies), was injected into C57BL/6J mouse zygotes that were transferred to recipient female mice. Microinjection was performed at the Biocenter Oulu Transgenic and Tissue Phenotyping Core Facility, University of Oulu, Oulu, Finland. Knockin-positive pups were identified by restriction digestion of a PCR product from the modified region and by Sanger sequencing. Founders carrying the mutations were mated with WT C57BL/6J mice, and F_1_ pups were analyzed in the same manner. Heterozygous founders were subsequently crossed with C57BL/6JCrl WT (Charles River) mice to establish the mutant strain. For analysis of homozygous embryos, heterozygous mice were crossed together and embryos harvested at E15.5 or E18.5.

### Immunofluorescence staining.

For whole-mount staining, embryonic dorsal skins and mesenteries were fixed with 4% paraformaldehyde (PFA) for 1 hour at room temperature, washed with PBS, permeabilized with 0.3% Triton X-100 in PBS, blocked with donkey immunomix (5% normal donkey serum, 0.2% BSA, 0.3% Triton X-100, and 0.05% sodium azide, all in PBS) (5% donkey serum, 0.2% BSA, 0.05 % NaN_3_, and 0.3% Triton X-100 in PBS) for 2 hours at room temperature, and incubated with primary antibodies diluted in blocking buffer for 1–3 days at +4°C. After incubation with the primary antibodies goat anti–mouse VEGFR3 (AF743, R&D Systems), rabbit anti–mouse Prox1 (AngioBio 11-002P), and rabbit anti–mouse LYVE1 (75), samples were washed with washing solution (0.3% Triton X-100 in PBS) and subsequently incubated with Alexa Fluorochrome–conjugated secondary antibodies diluted in washing solution overnight at +4°C. The samples were washed with washing solution, postfixed with 4% PFA, and washed with PBS, followed by mounting with Vectashield mounting medium (Vector Laboratories).

For cell surface immunofluorescence staining, cells grown on glass coverslips were washed with ice-cold PBS, fixed with 4% PFA, blocked with 3% BSA in PBS, and incubated with anti-TIE1 antibody (AF619, R&D Systems) for 1 hour. The cells were then washed for 10 minutes with PBS twice, incubated with Alexa Fluorochrome–conjugated secondary antibodies and DAPI, and mounted in Vectashield mounting medium (Vector Laboratories). For total TIE1, the staining protocol was the same except that after fixation, cells were permeabilized with 0.1% Tx-100 in PBS for 5 minutes and washed twice with PBS before proceeding to blocking.

### Western blotting of embryonic lung lysates.

Embryonic lungs were lysed in tissue lysis buffer (10 mM Tris-HCl, 140 mM NaCl, 1% Tx-100, 0.1% sodium deoxycholate, 0.1% SDS, 0.5 mM EGTA, 1 mM PMSF, protease inhibitor cocktail [P8340, Sigma-Aldrich], 20 mM β-glycerophosphate, 100 mM NaF, 5 mM sodium pyrophosphate, and 5 mM Na_3_VO_4_) using high-density zirconium oxide beads (MB2Z015, Biotop) and PowerLyzer 24 tissue homogenizer (MoBio Laboratories). Insoluble tissue debris was removed by centrifugation. Fifteen micrograms of total lysates were separated in SDS-PAGE, or lysate containing 500–1,000 μg of total protein was used for immunoprecipitation. Goat anti-TIE1 (AF619, R&D Systems), goat anti-VEGFR3 (AF743, R&D Systems), goat anti-PROX1 (AF2727, R&D Systems), and mouse anti-HSC70 (sc-7298, Santa Cruz Biotechnology) antibodies were used for immunoblotting. Experiments were repeated 3 times for TIE1-R979W and twice for TIE1-M1106R embryos.

### Image acquisition and quantification.

Confocal images were acquired using an Andor Dragonfly 505 spinning disc confocal microscope and a Leica Stellaris 8 FALCON/DLS confocal microscope. Images represent maximum-intensity projections of *Z*-stacks of single tiles or stitched multiple tile scans. Lymphatic vessel area in embryonic dorsal skins was quantified using AutoTube (76). Fluorescence staining intensity of TIE1 was quantified using ImageJ. Mean TIE1 signal intensity values were measured from cells that were completely visible in images, and surface TIE1 to total TIE1 signal ratio was calculated from 3–6 images per group.

### Statistics.

Data are expressed as mean ± SEM, and statistical evaluation was performed using either ordinary or Brown-Forsythe 1-way ANOVA, followed by Dunnett’s or Tukey’s post hoc test for multiple comparisons, using GraphPad Prism version 6.00, 9.00, or 10.00 for Mac OSX (GraphPad Software). *P* values of less than 0.05 were considered statistically significant.

### Study approval.

Written informed consent was received from all patients prior to participation in the study, as approved by Comité d’Ethique Hospitalo-Facultaire Saint-Luc — UCLouvain (B403201629786), as well as the local boards of the referring clinicians. Written informed consent was also received for the use of the photographs, and the record of informed consent has been retained. All experimental procedures involving mice, including generation of gene-modified knockin mouse lines using CRISPR/Cas9, were approved by the Project Authorisation Board, Regional State Administrative Agency of Southern Finland (license for CRISPR/Cas9: ESAVI/33743/2019).

### Data availability.

Ethics regulations in Belgium do not allow sharing of the whole-exome sequencing data. However, the coordinates of the variants provided in Table 1 clearly identify them in chromosomal positions according to the widely used *hg38* reference genome. Quantification data are provided in the Supporting Data Values file.

## Author contributions

PB performed the genetic analyses and identified the variants. LP characterized the stop codon variant with PB, and performed the studies in HEK293T cells. AM, VML, and MH characterized the variant structures, and AM, VML, MH, and AA characterized the variants in biochemical, cell biological signal transduction experiments, established the mutant mouse models and characterized them, and acquired and interpreted the results. MHS, RH, and LE designed and produced the knockin mouse models. SM, LMB, NR, AG, and IQ recruited the patients and performed detailed clinical explorations. KA and MV initiated and supervised the studies, and obtained funding to perform them. The manuscript was written by PB, AM, and VML, with the help of KA and MV. The content was approved by all coauthors. The authorship order among the 3 co–first authors was established based on (a) the identification of the mutations by PB, who also supervised LP; (b) the characterization of the mouse mutant models by AM, who also supervised MH; and (c) the structural and biochemical characterization of the variants by VML.

## Supplementary Material

Supplemental data

Unedited blot and gel images

Supporting data values

## Figures and Tables

**Table 1:**
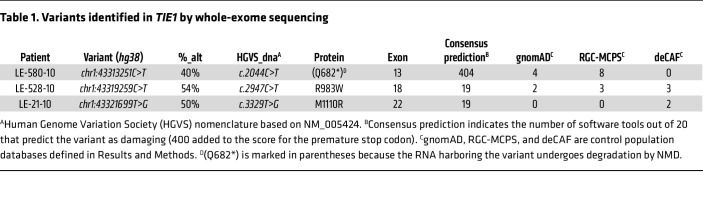
Variants identified in *TIE1* by whole-exome sequencing

## Version 1 05/31/2024

In-Press Preview

## Version 2 07/15/2024

Electronic publication
