# Supplementary material for: Loss-of-function mutations of the TIE1 receptor tyrosine kinase cause late-onset primary lymphedema
Source: J Clin Invest. 2024 May 30;134(14):e173586. doi: 10.1172/JCI173586 (PMC11245153; doi:10.1172/JCI173586)
Supplement: Supplemental data [file jci-134-173586-s076.pdf]

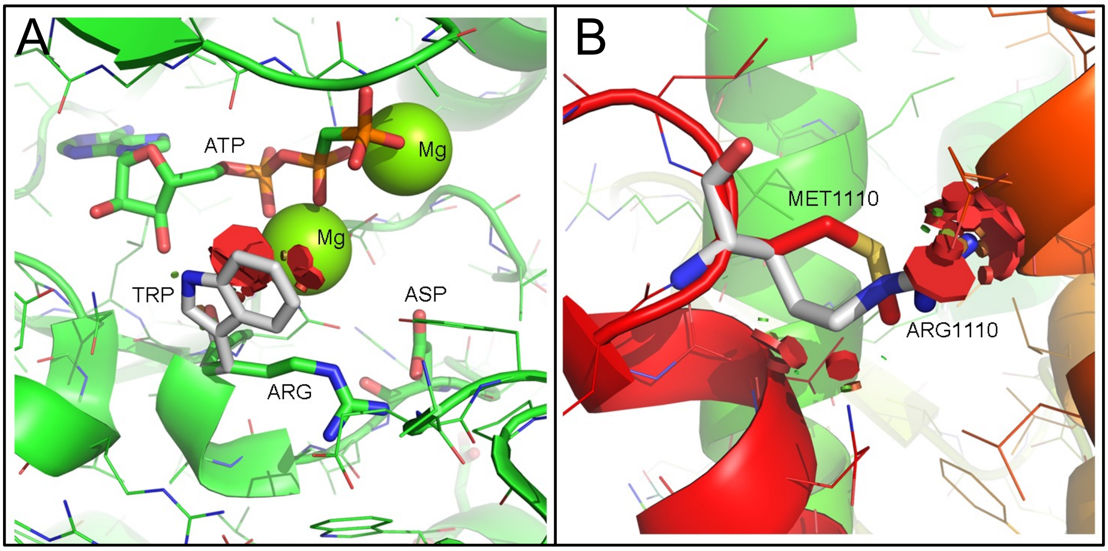

Supplemental Figure S1. Structural characterization of the two TIE1 pathogenic missense variants. (**A**) Tryptophan mutant in the position of Arg983 in the ATP-binding pocket of the homologous FGFR2 kinase domain. (**B**) Arginine in the position of Met1110. The side chains of the mutant residues are shown in the most common rotamer, and steric clash with the rest of the model in this orientation is shown with the red discs.

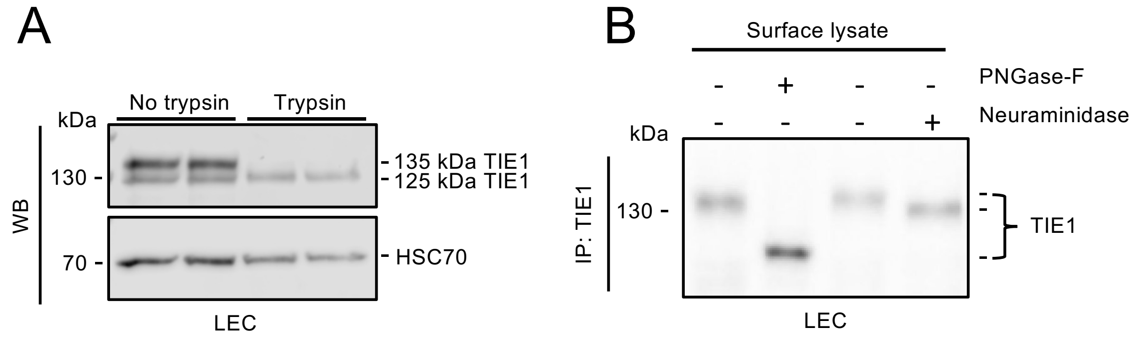

Supplemental Figure S2. Effect of trypsin, PNGase-F and Neuraminidase on cell surface TIE1 in LEC lysates. (**A**) Western blot analysis of TIE1 in control or trypsin-treated LECs. (**B**) Western blot analysis of PNGase-F or neuraminidase (also called sialidase)-treated immunoprecipitates of LEC surface-TIE1.

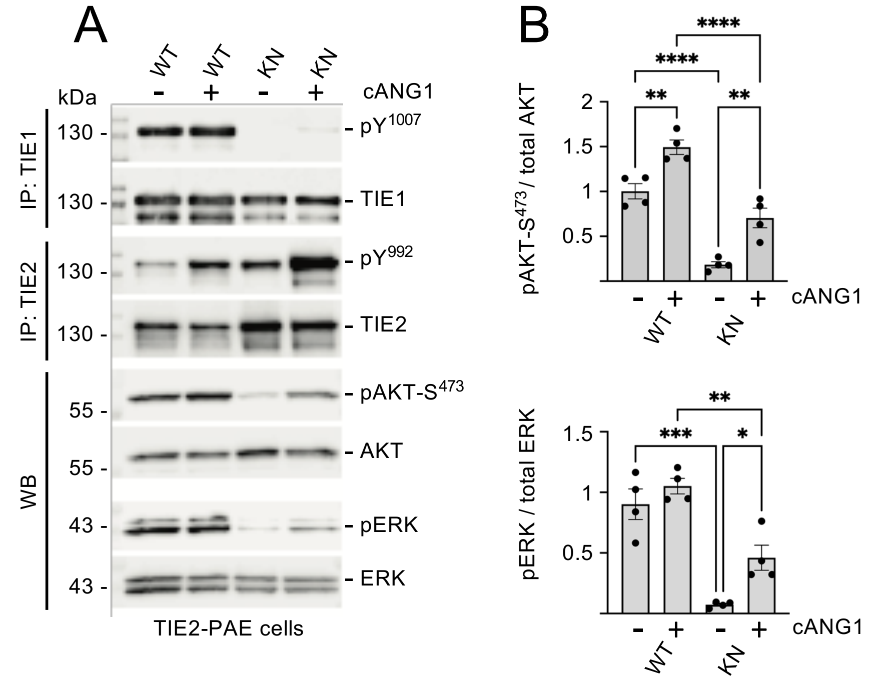

Supplemental Figure S3. Comparison of activation and downstream signaling by WT-TIE1 and its targeted kinase-negative (KN) mutant in ECs. (**A**) Western blot analysis of the cANG1-stimulated TIE2-PAE cells transduced with WT or KN (KN; ref 55)-TIE1. (**B**) Quantification of phospho-AKT^{S473} and p-ERK and total AKT or ERK ratios in the cANG1-stimulated and unstimulated samples from **A**. Data shown as mean ± SEM, n=4, 1-way ANOVA with Tukey's post hoc test for multiple comparisons. * p< 0.05, **p< 0.01, ***p<0.001, **** p< 0.0001.

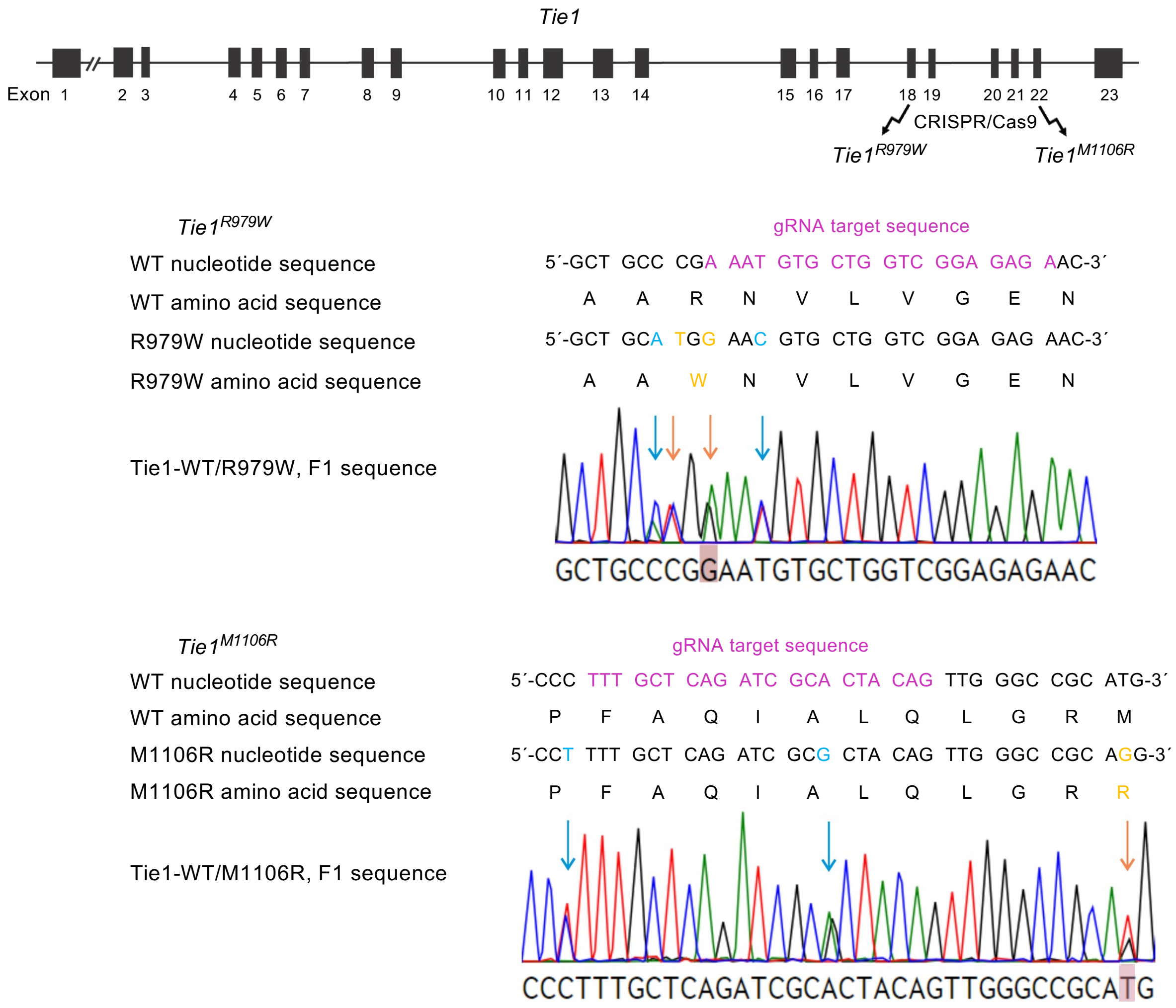

Supplemental Figure S4. Targeting strategy of the *Tie1* knock-in mouse lines. CRISPR/Cas9-based technology was used to create point mutations in exon 18 of mouse *Tie1* gene, resulting in R to W change in codon 979 (editions marked with orange) of the corresponding protein (TIE1-R979W). Similarly, exon 22 was targeted to create point mutation resulting in M to R change in codon 1106 (edition marked with orange) of TIE1 protein (TIE1-M1106R). Silent mutations (marked with blue in the KI nucleotide sequence) were included in the repair template to remove the PAM site and to create a new restriction enzyme cut site which was used for genotyping. Created point mutations are highlighted with arrows in the electropherograms from Sanger sequencing of *Tie1*$^{WT/R979W}$ and *Tie1*$^{WT/M1106R}$ F1 mice.

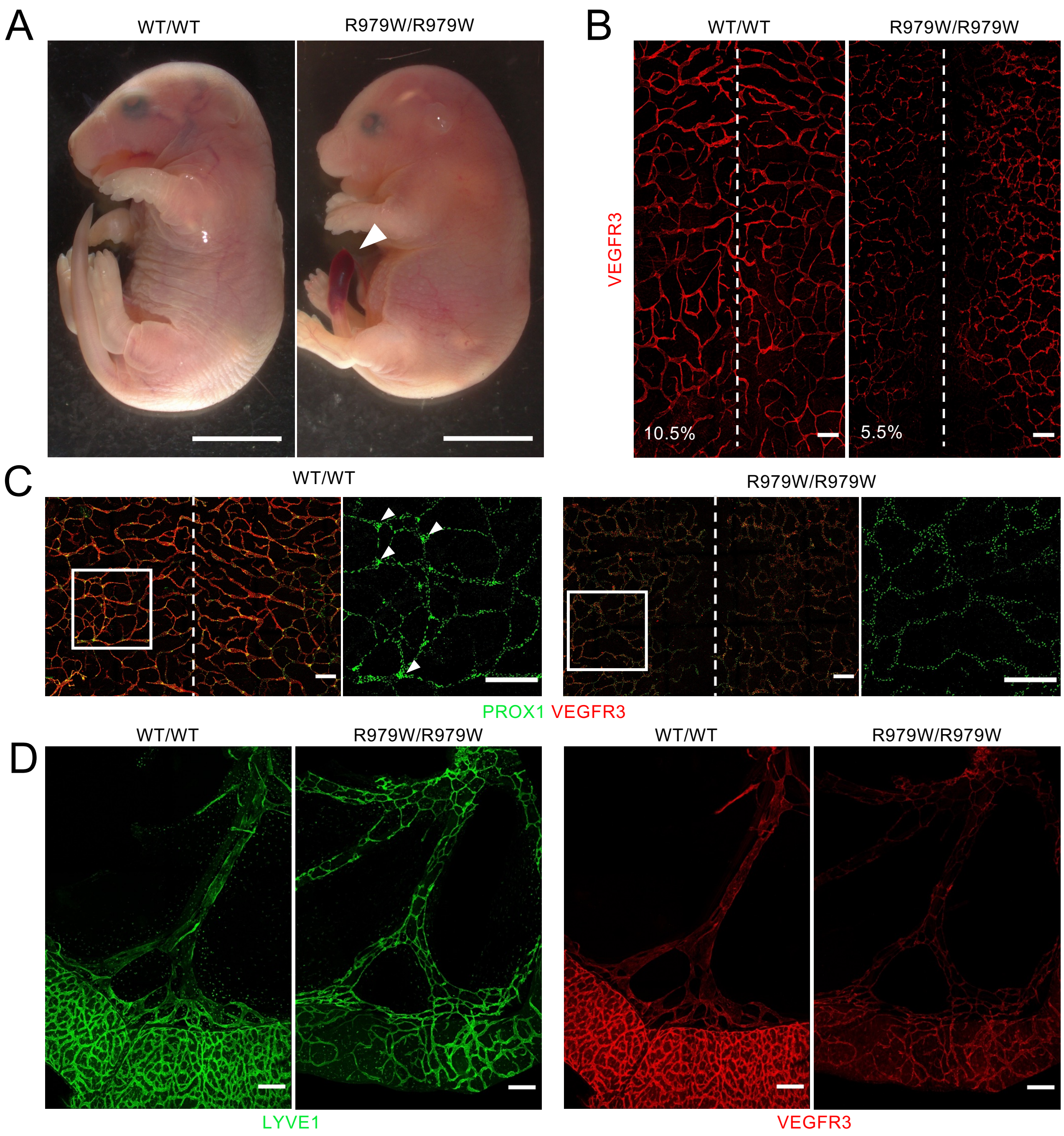

Supplemental Figure S5. Swelling, delayed lymphatic outgrowth, immature collecting vessels and lack of valves in E18.5 homozygous *Tie1*^{R979W} embryos. (**A**) Macroscopic images of *Tie1*$^{WT/WT}$ and mutant *Tie1*$^{R979W/R979W}$ embryos at E18.5. White arrowhead points to hemorrhage at the tip of the tail. Scale bars: 5 mm. (**B**) VEGFR3 staining in E18.5 dorsal skin. Dashed line indicates the dorsal midline. Scale bars: 300 μm. Quantifications (%) of lymphatic vessel area around the dorsal midline are marked in white. **(C)** VEGFR3 and PROX1 staining in dorsal skin. The dashed line indicates the dorsal midline. The boxed areas in **C** are shown magnified on the right with PROX1 staining only. White arrowheads point to lymphatic valves. Scale bars: 300 μm. (**D**) Staining of LYVE1 and VEGFR3 in developing gut and mesenterium. Note the delayed LYVE1 downregulation, the pattern of the mesenterial vessels and weaker expression of VEGFR3 in the homozygous mutant mice. Scale bars: 300 μm.

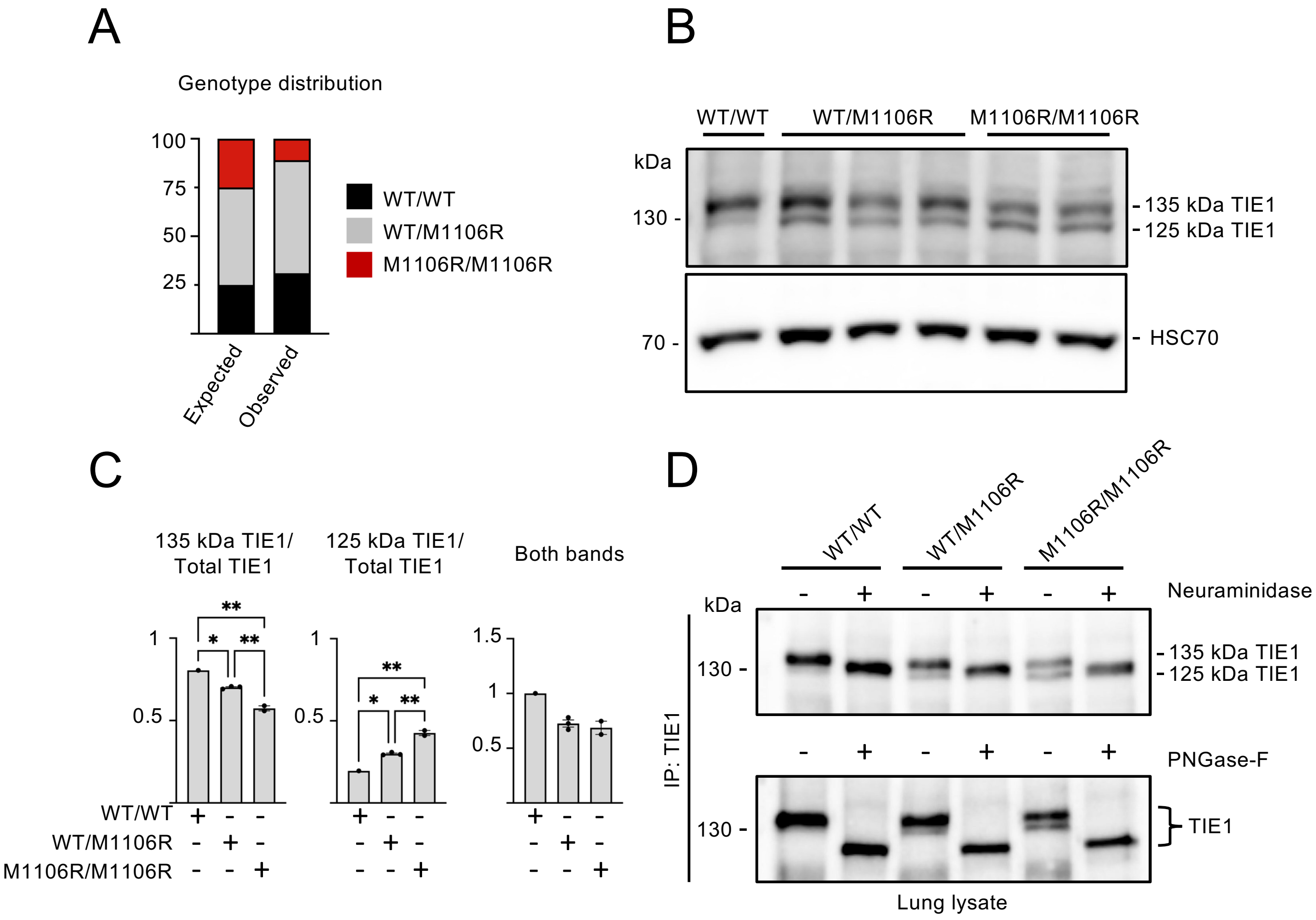

Supplemental Figure S6. Postnatal lethality and altered ratio of cell-surface vs intracellular TIE1 in E18.5 *Tie1^{M1106R}* mutant embryos. (**A**) Percentages of the indicated genotypes among pups born from heterozygous *Tie1$^{WT/M1106R}$* matings. n=9 litters (observed WT/WT n=20, WT/M1106R n=38, M1106R/M1106R n=7). (**B**) Western blot analysis of TIE1 in lung lysates from WT, heterozygous (*Tie1$^{WT/M1106R}$*) and homozygous (*Tie1$^{M1106R/M1106R}$*) embryos. (**C**) Quantification of the proportions of cell-surface 135 kDa and intracellular 125 kDa TIE1 polypeptides relative to total TIE1 from the Western blots in **B**. The graph "Both bands" is the Total TIE1 normalized to HSC70. n=2 independent experiments. Statistical analysis was done using ordinary 1-way ANOVA with Tukey's post-hoc test for multiple comparisons. Data shown as mean ± SEM. *p< 0.05, **p< 0.01. (**D**) Western blot analysis of WT, heterozygous (*Tie1$^{WT/M1106R}$*) and homozygous (*Tie1$^{M1106R/M1106R}$*) adult mouse TIE1 lung immunoprecipitates treated with neuraminidase or PNGase-F.
