# Supplementary material for: Loss-of-function mutations of the TIE1 receptor tyrosine kinase cause late-onset primary lymphedema
Source: J Clin Invest. 2024 May 30;134(14):e173586. doi: 10.1172/JCI173586 (PMC11245153; doi:10.1172/JCI173586)

Full unedited gels for Figure 3A

The parts shown in the Figure are delineated by a blue box

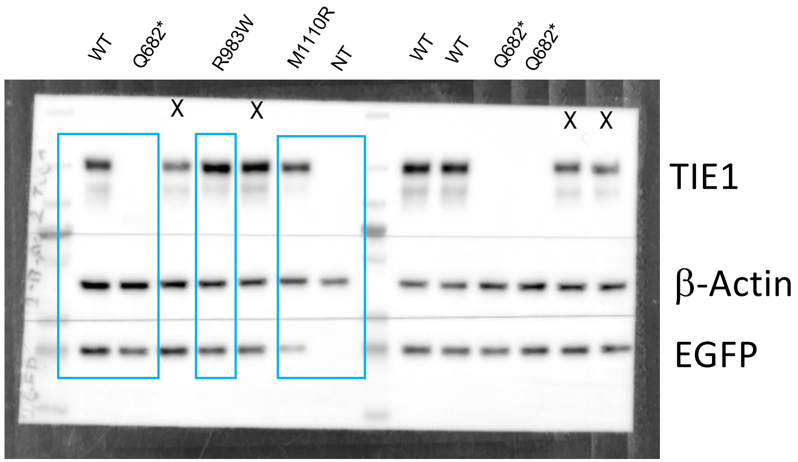

20 second exposure

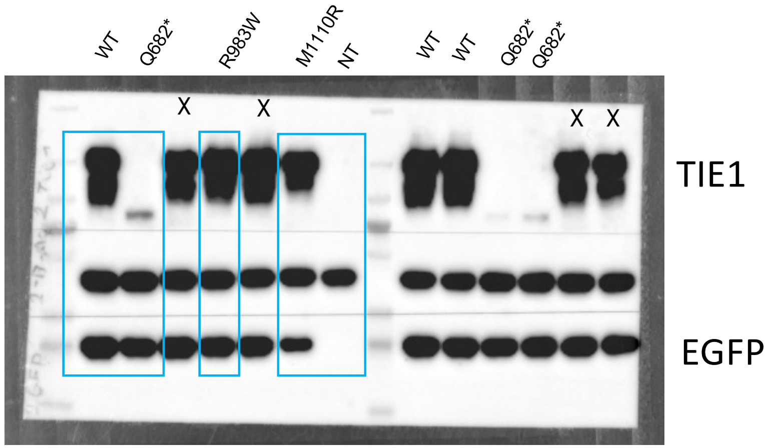

5 minute exposure

Full unedited gels for Figure 3B

The parts shown in the Figure are delineated by a blue box

Supernatants

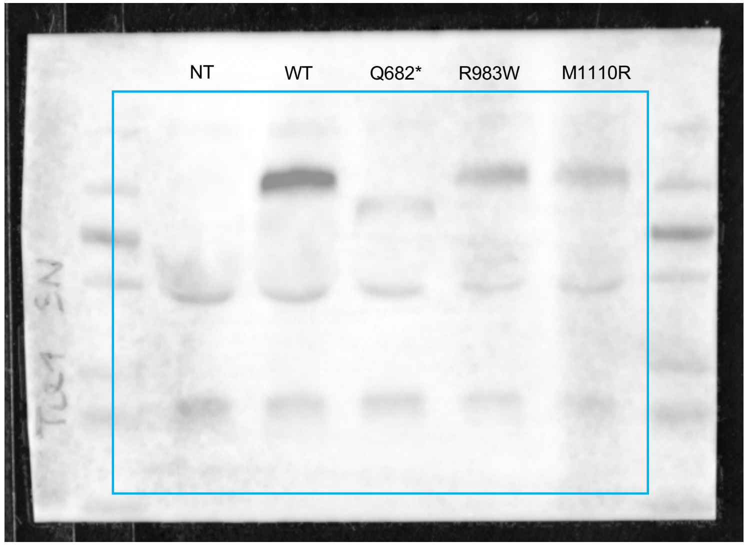

Blot: TIE1

20 second exposure

Full unedited gels for Figure 4A

The parts shown in the Figure are delineated by a blue box

Strep-Tactin pull-down

Blot: pY (4G10) (Merck 05-321)

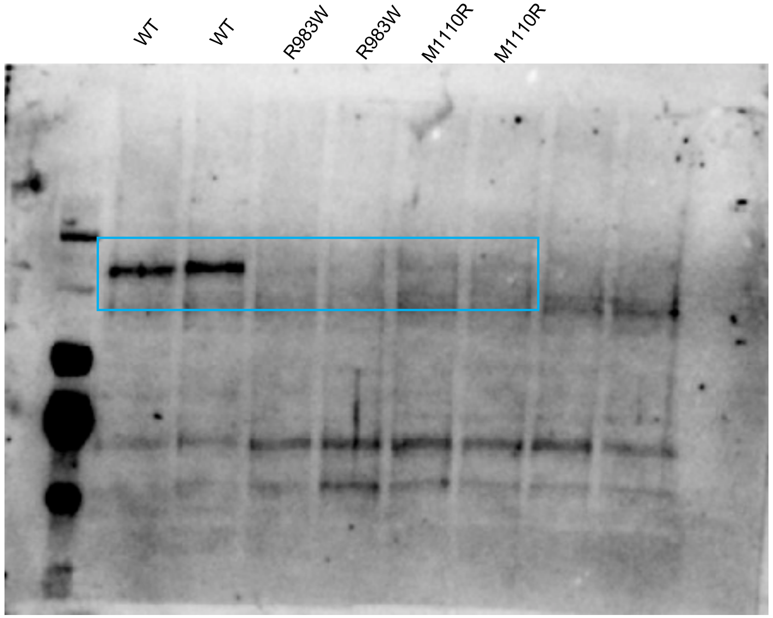

Blot: a-hTIE-1
(AF619)
(R&D Systems)

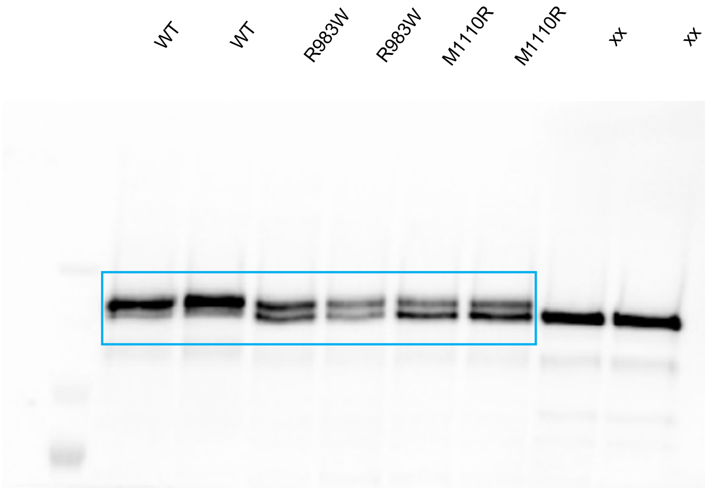

Full unedited gels for Figure 5A

The parts shown in the Figure are delineated by a blue box

Upper images

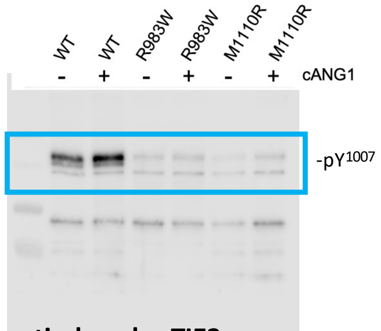

anti-phospho-TIE2 (pY-992)
(AF2720, R&D Systems)

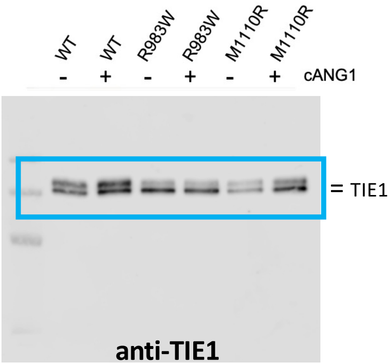

anti-TIE1
(AF619, R&D Systems)

This antibody recognizes pY1007 on TIE1

Bottom images

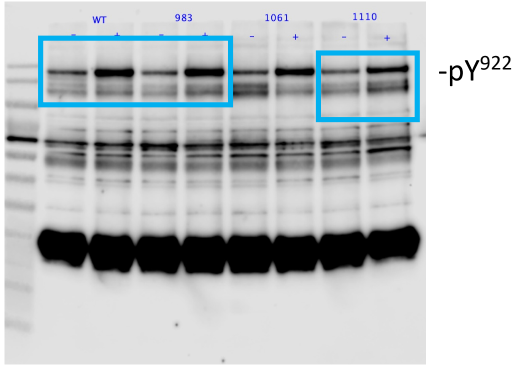

anti-phospho-TIE2 (pY-992)
(AF2720, R&D Systems)

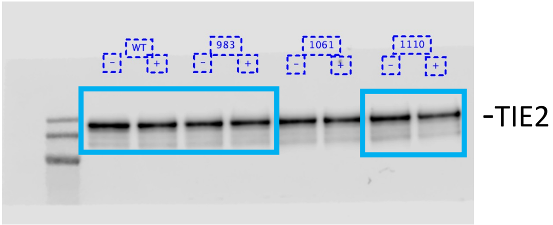

anti-TIE2
(AF313, R&D Systems)

Full unedited gels for Figure 5C

The parts shown in the Figure are delineated by a blue box

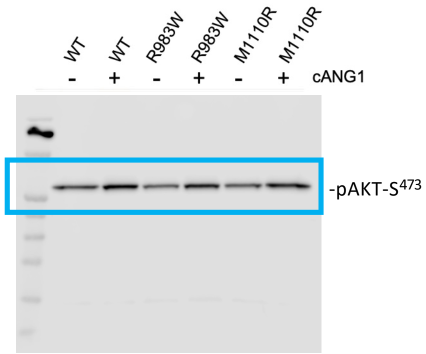

Phospho-AKT (S473)
(9271L, Cell Signaling)

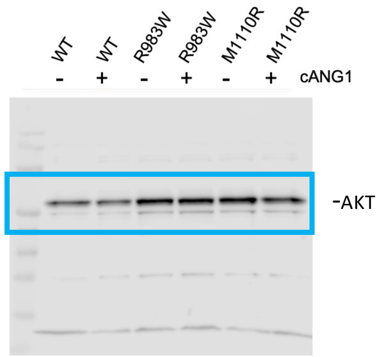

Total-AKT
(9272, Cell Signaling)

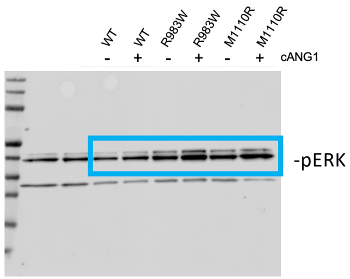

Phospho-ERK
(9106, Cell Signaling)

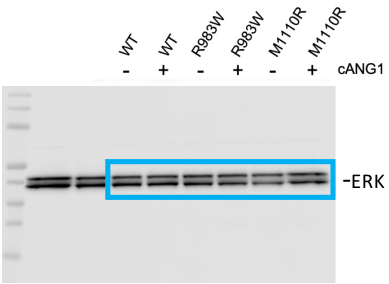

Total-ERK
(9102L, Cell Signaling)

Continued on next page ...

<u>**Full unedited gels for Figure 5C**</u>

The parts shown in the Figure are delineated by a blue box

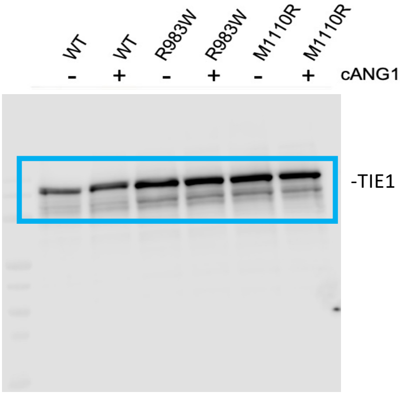

anti-TIE1
(AF619, R&D Systems)

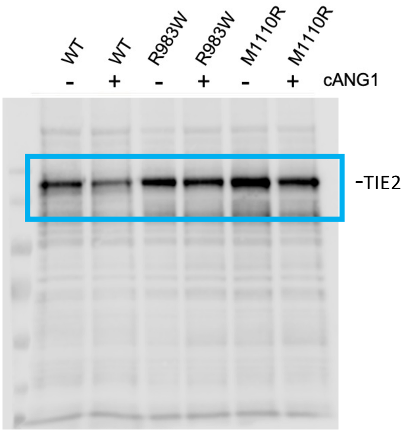

anti-TIE2
(AF313, R&D Systems)

Full unedited gels for Figure 6C

The parts shown in the Figure are delineated by a blue box

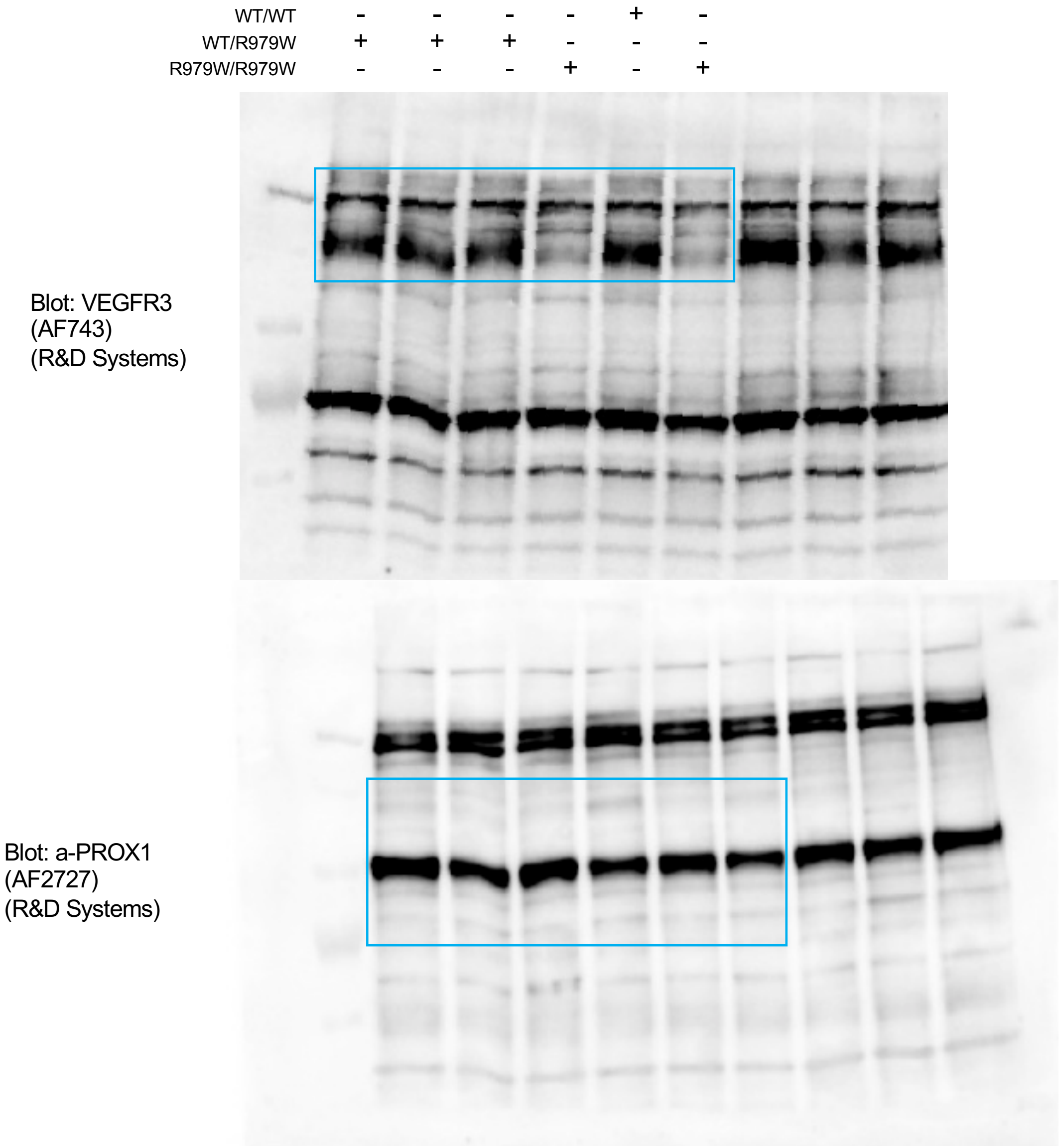

Full unedited gels for Figure 6D

The parts shown in the Figure are delineated by a blue box

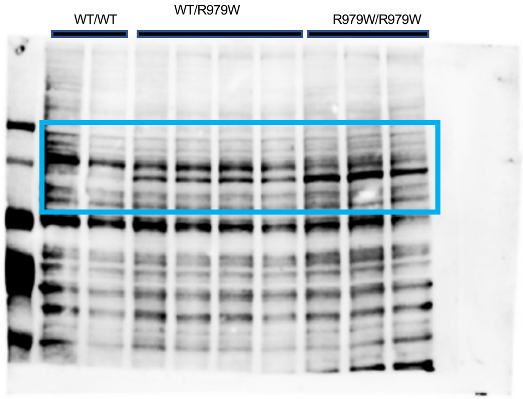

Blot TIE1
Antibody AF619
(R&D Systems)

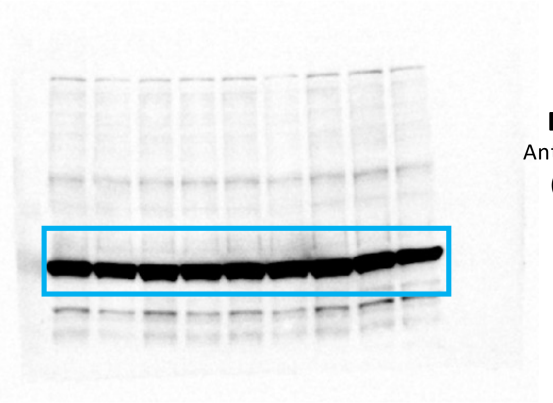

Blot HSC70
Antibody sc-7298
(Santa Cruz)

Full unedited gels for Figure S2A

The parts shown in the Figure are delineated by a blue box

Blot TIE1
Antibody AF619
(R&D Systems)

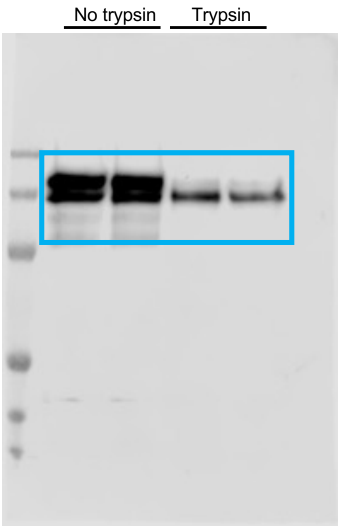

Blot HSC70
Antibody sc-7298 (Santa Cruz)

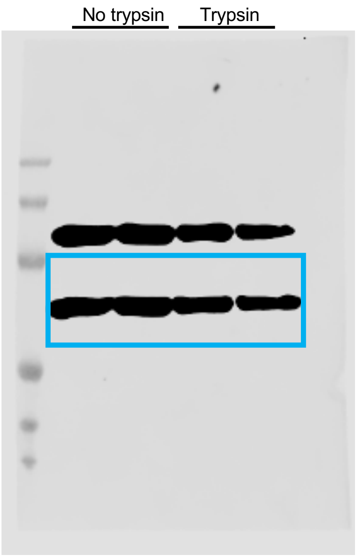

Full unedited gels for Figure S2B

The parts shown in the Figure are delineated by a blue box

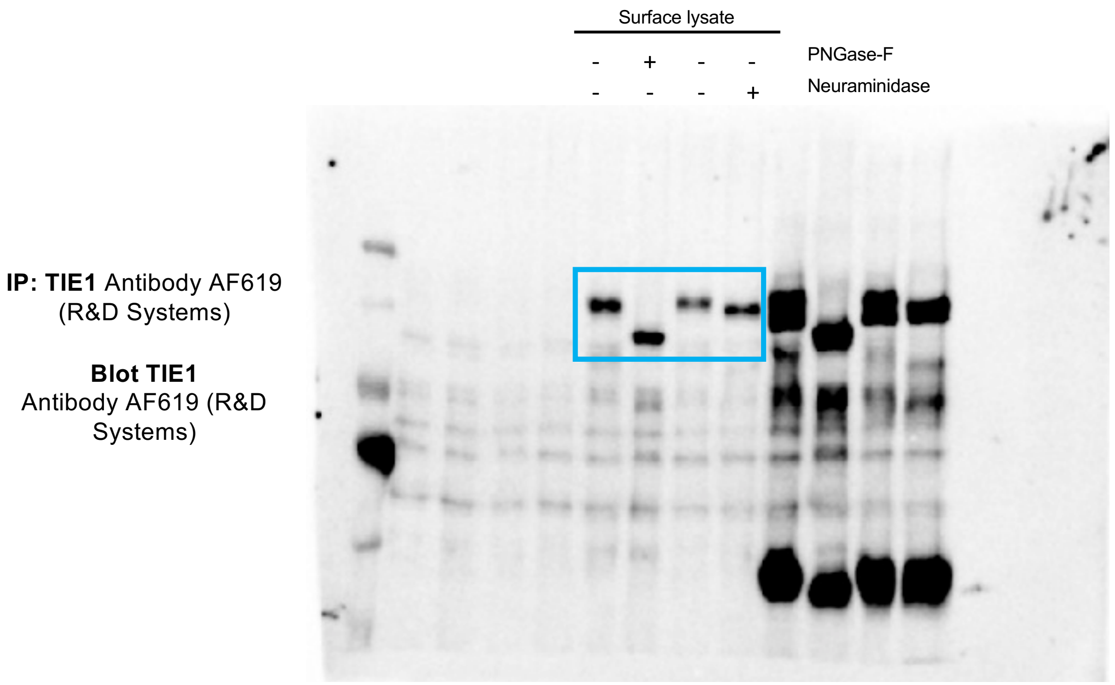

<u>Full unedited gels for Figure S3A</u>

The parts shown in the Figure are delineated by a blue box

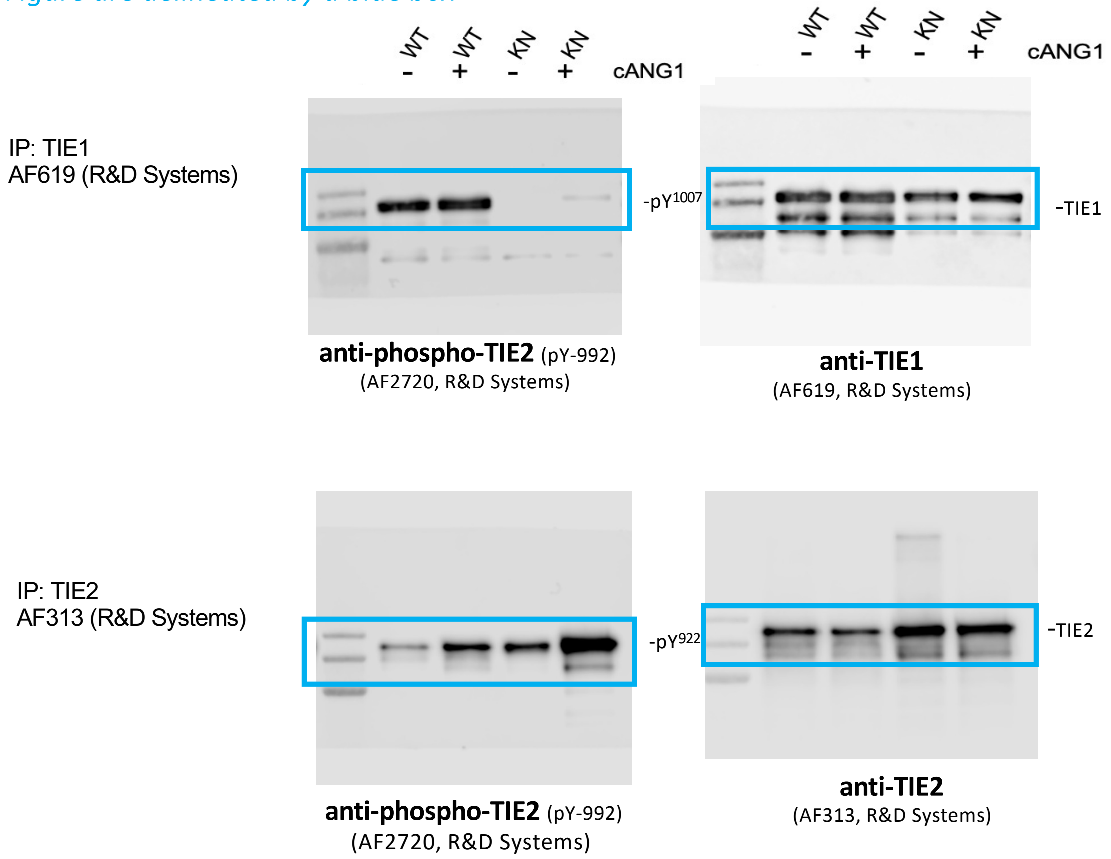

Continued on next page ...

Full unedited gels for Figure S3A

The parts shown in the Figure are delineated by a blue box

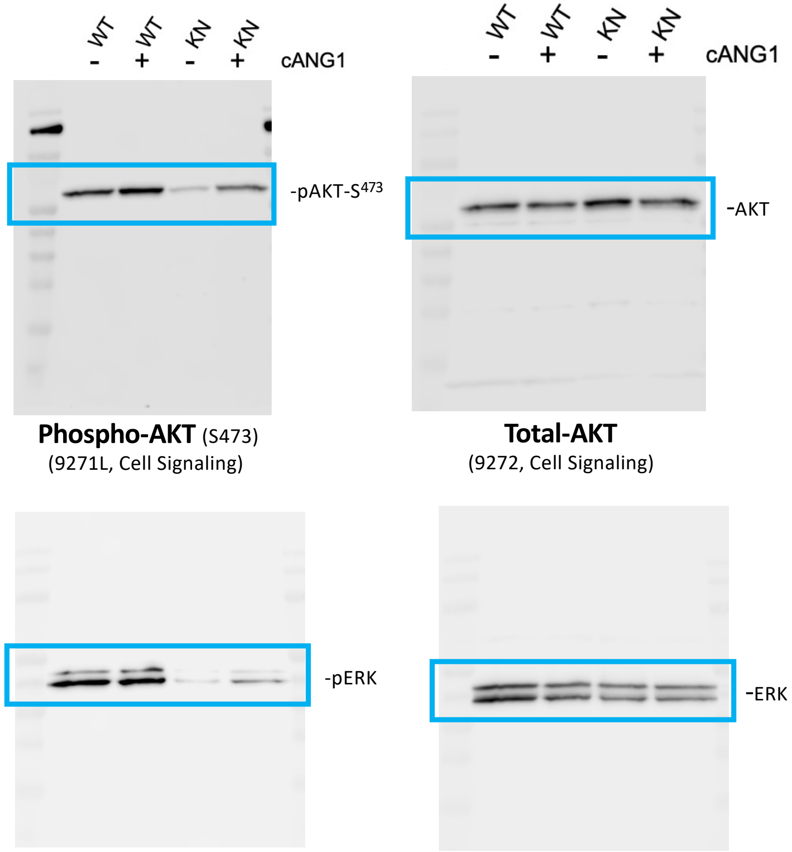

Phospho-AKT (S473)
(9271L, Cell Signaling)

Total-AKT
(9272, Cell Signaling)

Phospho-ERK
(9106, Cell Signaling)

Total-ERK
(9102L, Cell Signaling)

Full unedited gels for Figure S6B

The parts shown in the Figure are delineated by a blue box

Blot TIE1
Antibody AF619
(R&D Systems)

Blot HSC70
Antibody sc-7298
(Santa Cruz)

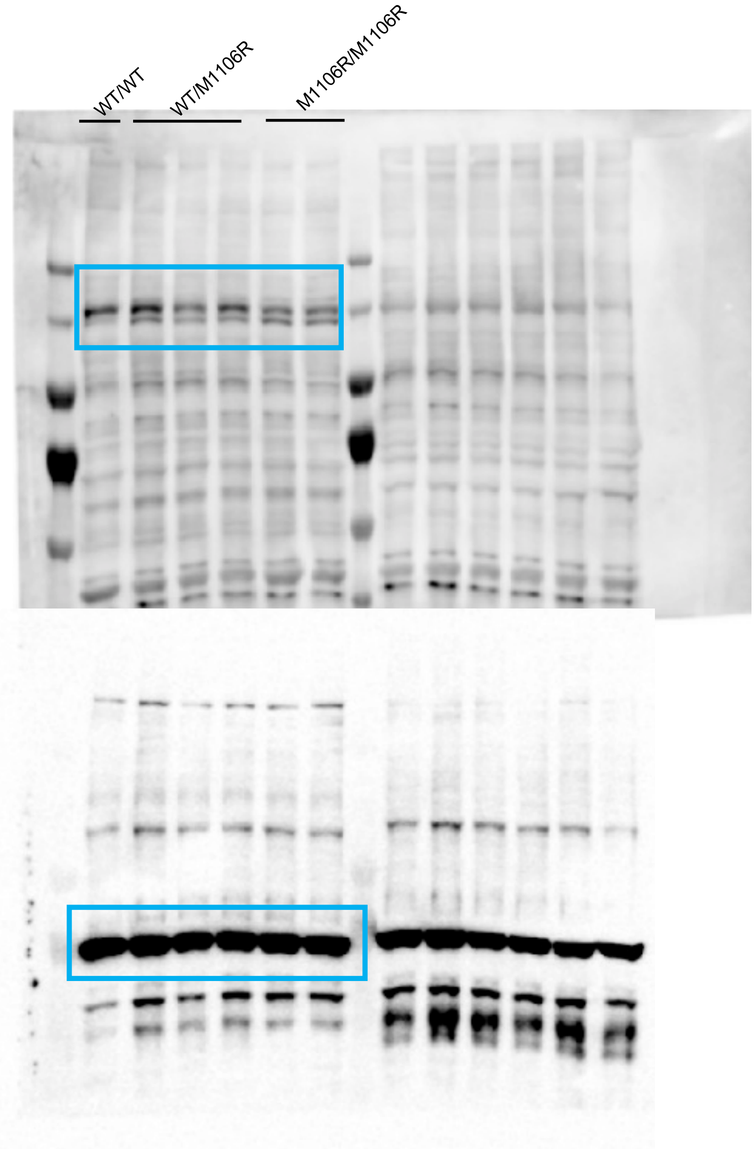

Full unedited gels for Figure S6D

The parts shown in the Figure are delineated by a blue box

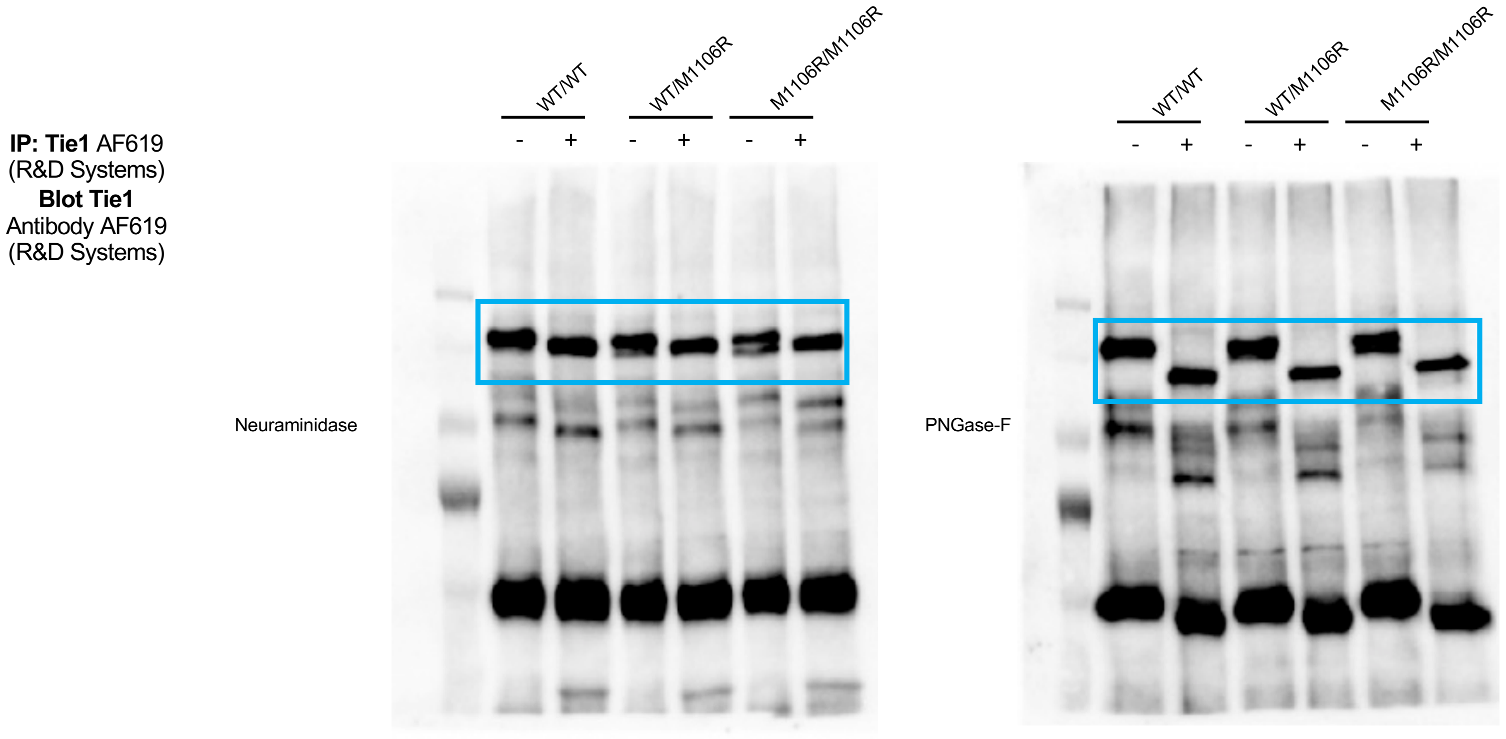

Supplement: Unedited blot and gel images [file jci-134-173586-s077.pdf]
